# A library of cancer testis specific T cell receptors for T cell receptor gene therapy

**DOI:** 10.1016/j.omto.2023.100738

**Published:** 2023-10-23

**Authors:** Marije A.J. de Rooij, Dennis F.G. Remst, Dirk M. van der Steen, Anne K. Wouters, Renate S. Hagedoorn, Michel G.D. Kester, Miranda H. Meeuwsen, Tassilo L.A. Wachsmann, Arnoud H. de Ru, Peter A. van Veelen, Els M.E. Verdegaal, J.H. Frederik Falkenburg, Mirjam H.M. Heemskerk

## Main text

(Molecular Therapy: Oncolytics *28*, 1–14; March 2023)

In the originally published version of this article, there was an error in Figure 4B. In the graph second from the top, MAGE-A1 LTQ/A1 is indicated in black, but instead this should be MAGE-A1 KVL/A2.

This error has been corrected in the figure online, and the authors apologize for any confusion this may have caused.

**Figure:**
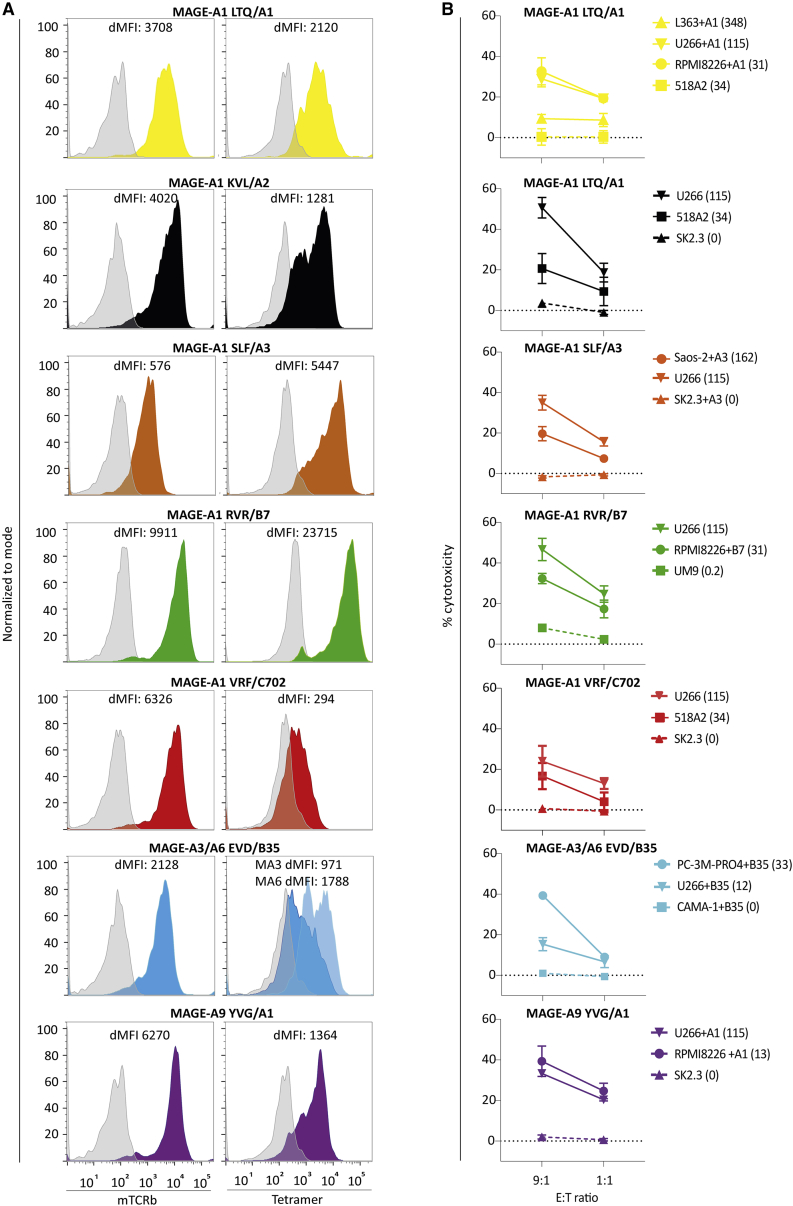
Figure 4. TCR expression and cytotoxicity of transduced primary CD8⁺ T cells against malignant cell lines (original)

**Figure:**
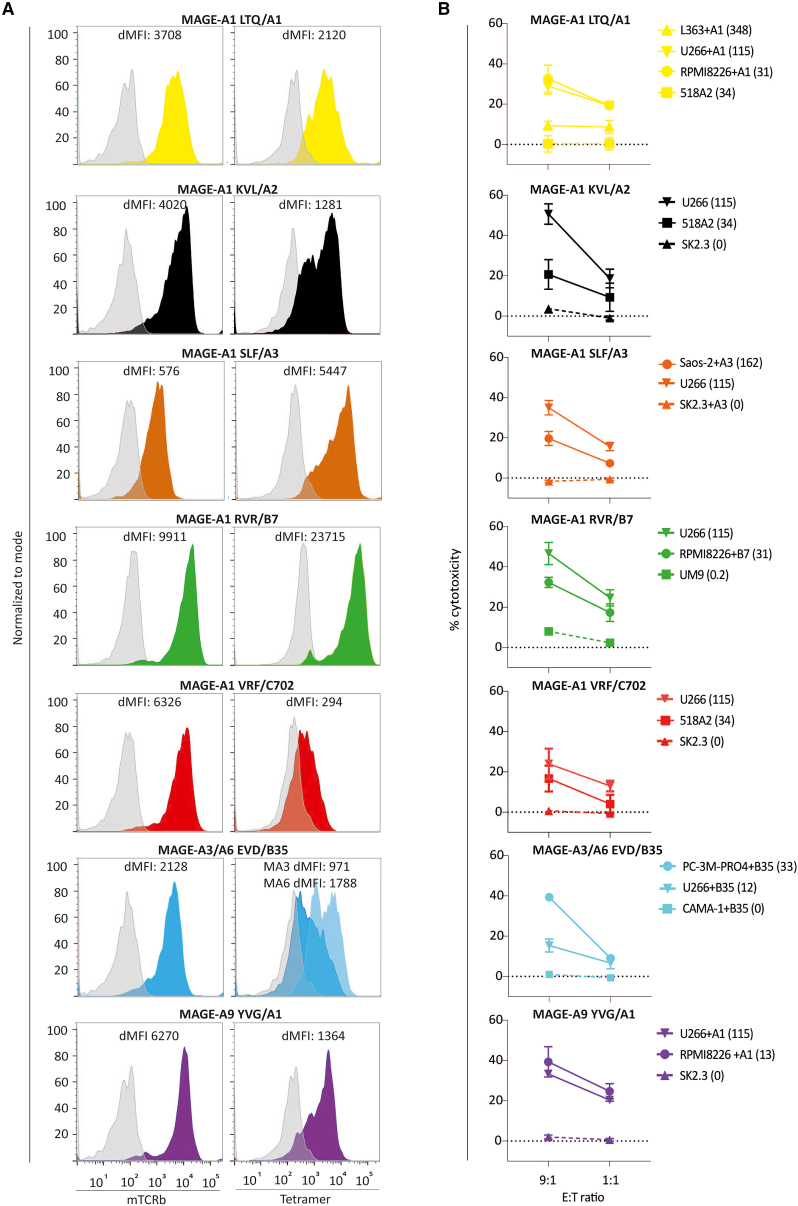
Figure 4. TCR expression and cytotoxicity of transduced primary CD8⁺ T cells against malignant cell lines (corrected)

