# Supplementary material for: A library of cancer testis specific T cell receptors for T cell receptor gene therapy
Source: Mol Ther Oncolytics. 2022 Dec 2;28:1–14. doi: 10.1016/j.omto.2022.11.007 (PMC9792401; doi:10.1016/j.omto.2022.11.007)
Supplement: Document S1. Figures S1–S6 and Tables S1–S3 [file mmc1.pdf]

OMTO, Volume *28*

Supplemental information

A library of cancer testis specific T cell receptors for T cell receptor gene therapy

Marije A.J. de Rooij, Dennis F.G. Remst, Dirk M. van der Steen, Anne K. Wouters, Renate S. Hagedoorn, Michel G.D. Kester, Miranda H. Meeuwsen, Tassilo L.A. Wachsmann, Arnoud H. de Ru, Peter A. van Veelen, Els M.E. Verdegaal, J.H. Frederik Falkenburg, and Mirjam H.M. Heemskerk

A

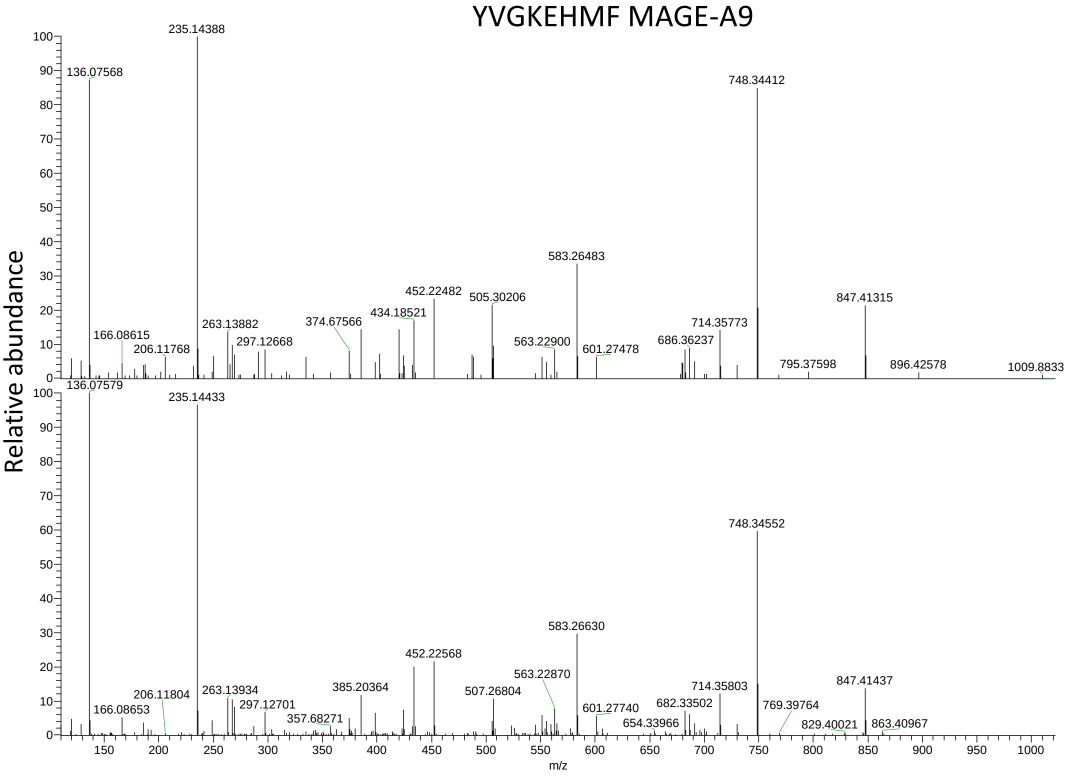

B

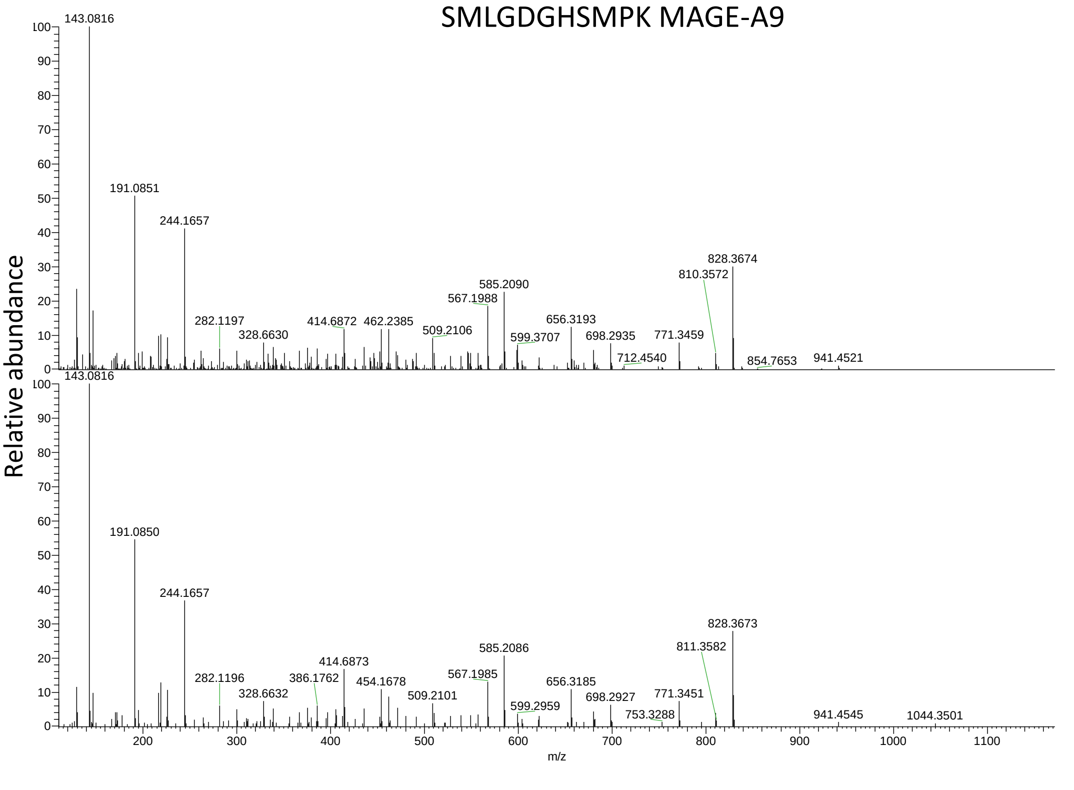

Figure S1. Peptide sequence validation of matching tandem mass spectra of eluted (top) and synthetically (bottom) generated peptides. Two examples of matching tandem mass spectra of (A) the YVGKEHMF peptide presented in HLA-A*24:02 and (B) the SMLGDGHSMPK peptide presented in HLA-A*03:01. Both depicted peptides are derived from MAGE-A9.

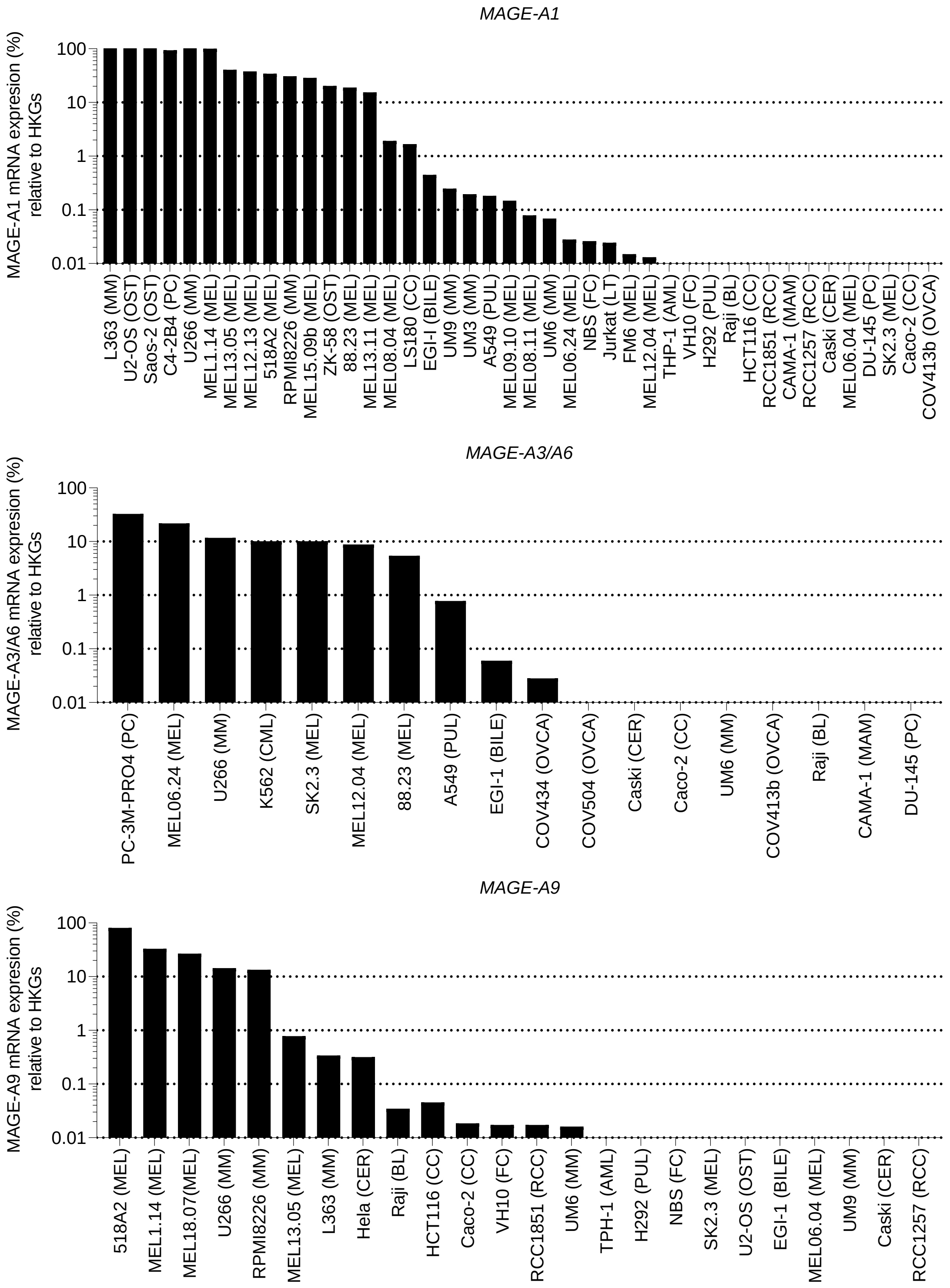

Figure S2. Target gene expression levels of *MAGE-A1*, *MAGE-A3/A6* and *MAGE-A9* mRNA levels. The expression levels were measured by qPCR for multiple cell lines with different origin including; multiple myeloma (MM), melanoma (MEL), prostate carcinoma (PC), fibroblast (FC), Burkitt lymphoma (BL), renal cell carcinoma (RCC), ovarium carcinoma (OVCA), colon carcinoma (CC), cervical carcinoma (CER, pulmonary carcinoma (PUL), leukemic T-cell lymphoblast (LT), bile duct carcinoma (BILE), osteosarcoma (OST), acute myeloid leukaemia (AML), chronic myeloid leukaemia (CML) cell lines and mammary carcinoma (MAM). Gene expression >0.01 is depicted in the graph relative to housekeeping genes (HKG). Values <0.01 relative gene expression are not depicted. *MAGE-A3* and *MAGE-A6* gene expression could not be discriminated from each other by qPCR.

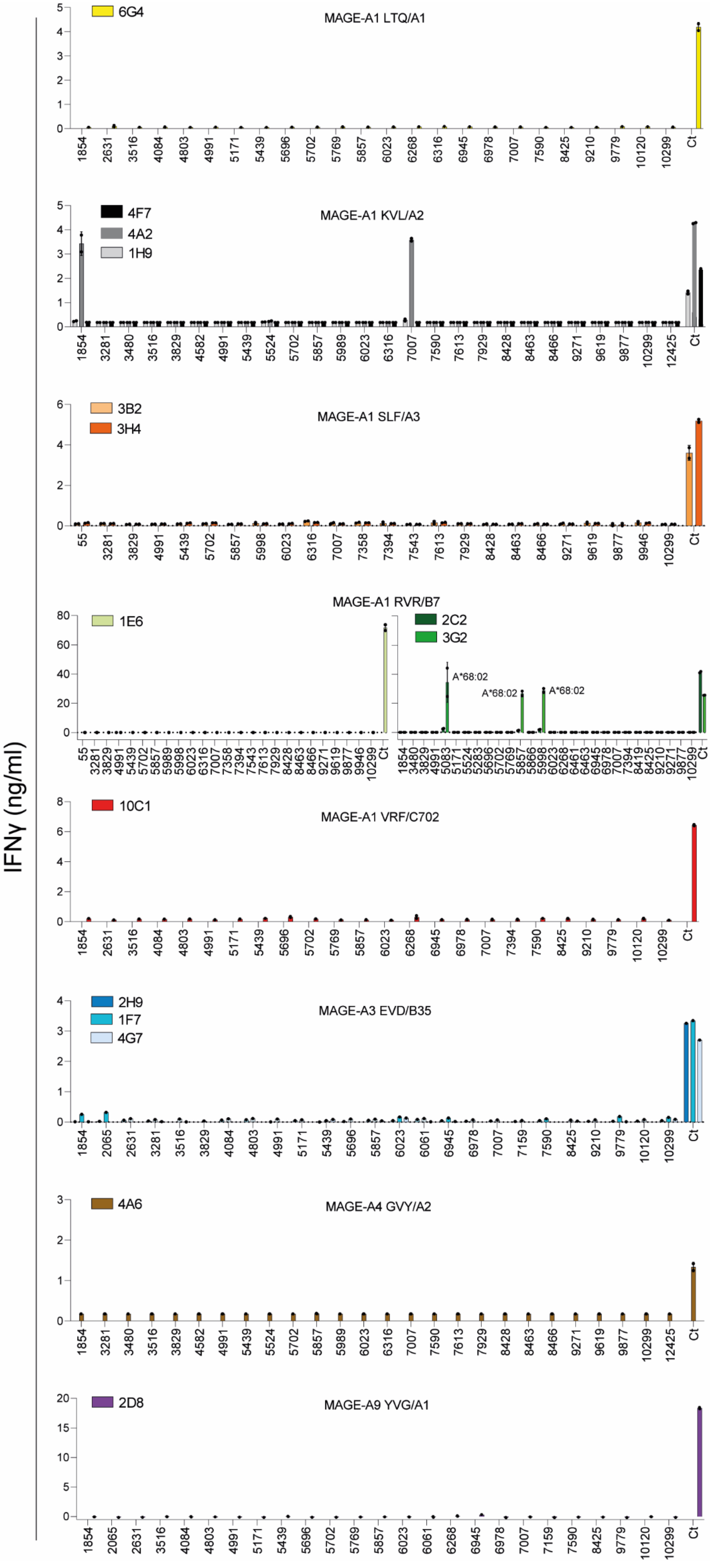
IFNγ (ng/ml)
MAGE-A1 LTQ/A1
6G4
MAGE-A1 KVL/A2
4F7
4A2
1H9
MAGE-A1 SLF/A3
3B2
3H4
MAGE-A1 RVR/B7
1E6
2C2
3G2
A*68:02
A*68:02
A*68:02
MAGE-A1 VRF/C702
10C1
MAGE-A3 EVD/B35
2H9
1F7
4G7
MAGE-A4 GVY/A2
4A6
MAGE-A9 YVG/A1
2D8
Ct

Figure S3. EBV-LCL panels to determine off-target reactivity against other peptide-HLA complexes. T cell clones were overnight stimulated with multiple EBV-LCLs. These EBV-LCL panels comprised of different EBV-LCLs expressing all HLA alleles with a prevalence of >1% in the Caucasian population. Peptide loaded HLA Td Raji cells were included as positive control (Ct) for T-cell function. The 4A2 clone (MAGE-A1 KVL/A2) demonstrated reactivity against 2 EBV-LCLs, the HLA restriction of this reactivity could not be identified, and therefore this T cell clone was excluded from further analyses. The 3G2 clone (MAGE-A1 RVR/B7) demonstrated reactivity against the HLA-A*68:02 positive EBV-LCLs. Values and error bars represent mean and standard deviations of technical duplicates.

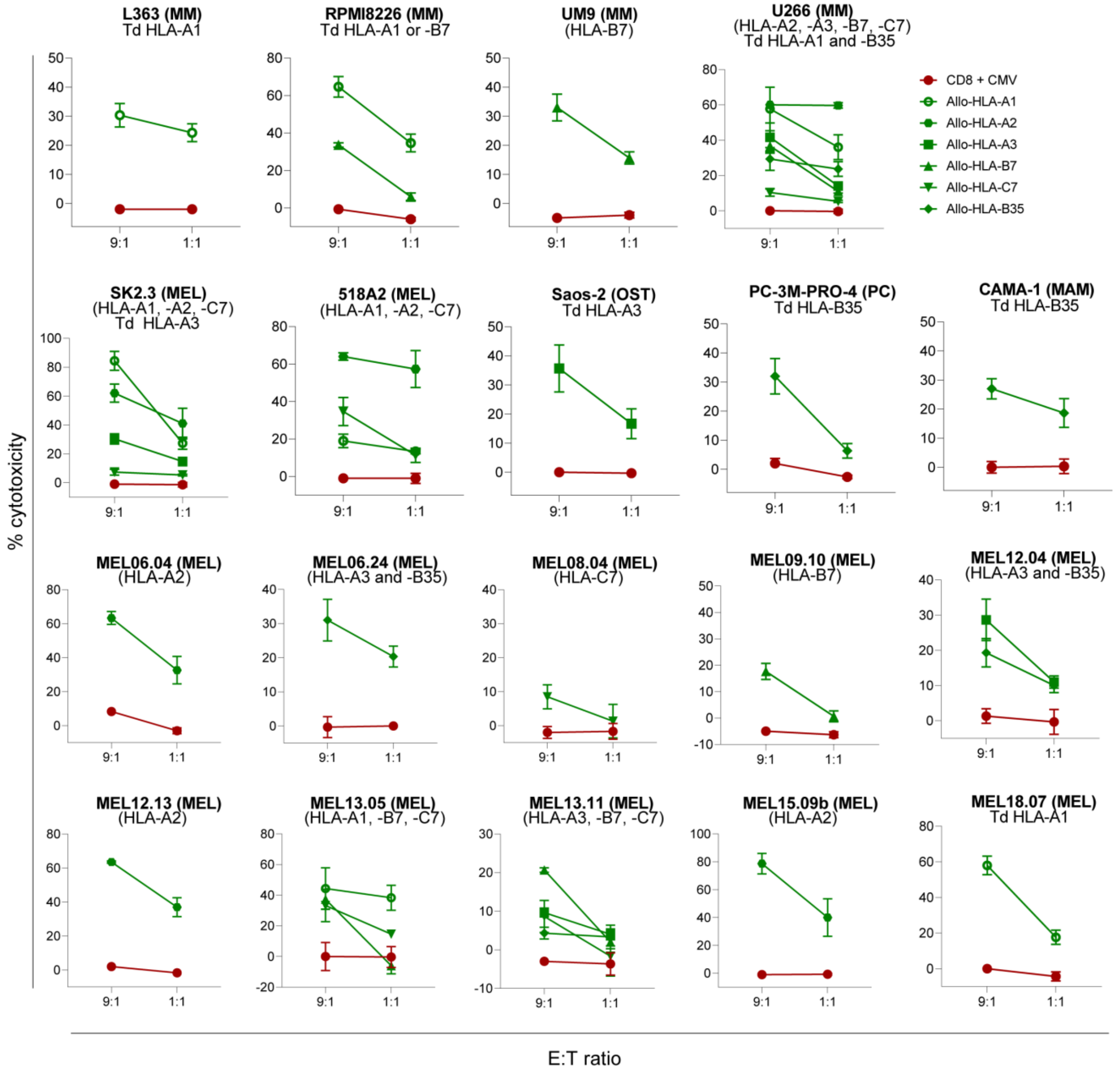

Figure S4. Positive and negative controls included for each target in the cytotoxicity assays. CMV TCR-T cell was included as a negative control (red) and an allo-HLA reactive T cell clone against the HLA of interest as a positive control (green). The tumor cell lines included were; Multiple myeloma (MM), melanoma (MEL), osteosarcoma (OST), prostate carcinoma (PC), and mammary carcinoma (MAM). Transduced HLA alleles are depicted in the graphs and naturally expressed HLA alleles are depicted between brackets. Cytotoxicity was assessed by an 6-hour ^{51}Cr-release assay with E:T ratio 9:1 and 1:1. Technical triplicates are depicted in the graph and the values are a representatives of at least two independent experiments.

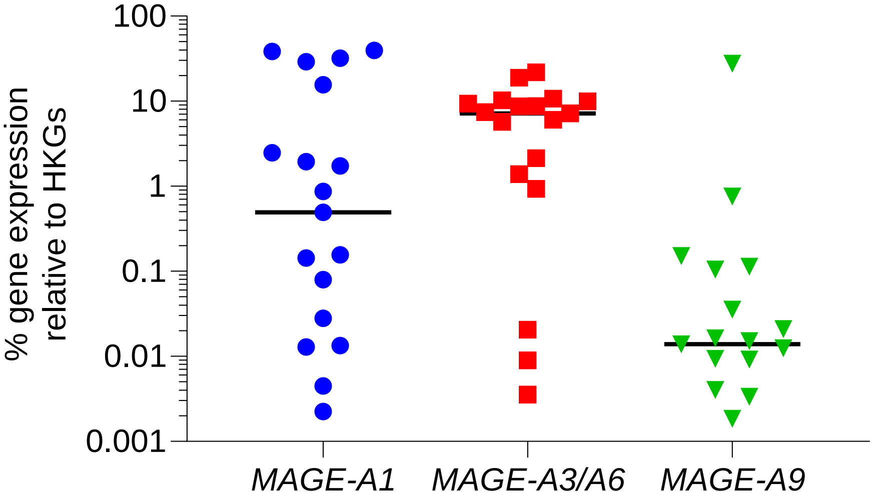

Figure S5. *MAGE-A1, MAGE-A3/A6* and *MAGE-A9* mRNA expression levels of early passage melanomas (passage ≤10) as measured by qPCR. *MAGE-A3* and *MAGE-A6* gene expression could not be discriminated from each other by qPCR. Gene expression >0.001% is depicted in the graph.

A

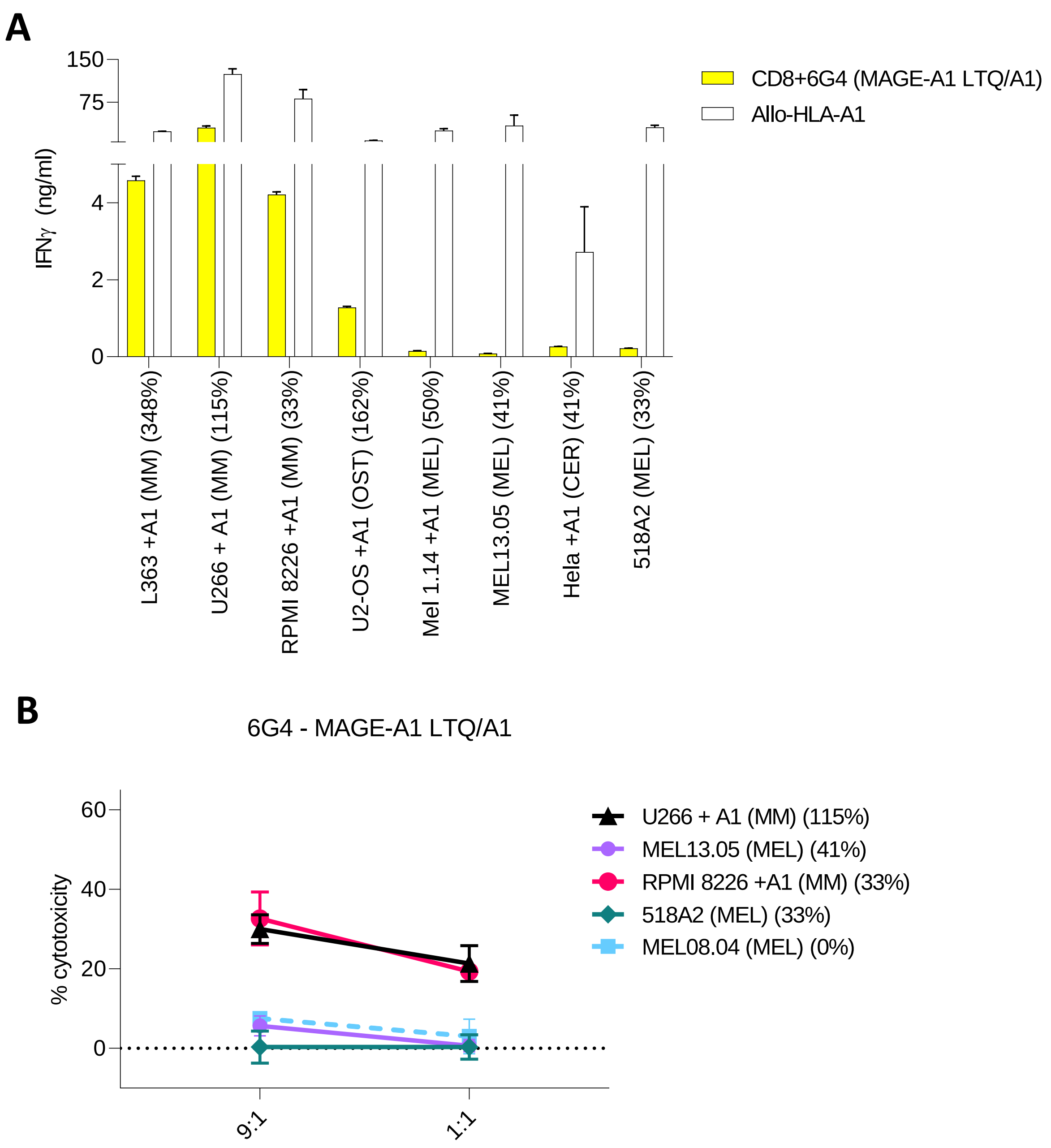

Figure S6. Reactivity of the 6G4 (MAGE-A1 LTQ/A1) TCR-T cells against tumor cell lines. (A) The 6G4 (MAGE-A1 LTQ/A1) TCR-T cell were overnight stimulated with MAGE-A1 expressing tumor cell lines of different origin including; melanoma (MEL), multiple myeloma (MM), cervical carcinoma (CER) and osteosarcoma (OST) cell lines. An allo-HLA reactive T cell clone was included to confirm proper HLA expression and recognition capacity of the targets. *MAGE-A1* gene expression levels, measured by qPCR, are depicted between brackets as percentage relative to housekeeping genes. Recognition was determined by IFN-γ production as measured by ELISA. Values and error bars represent mean and standard deviations of technical duplicates. (B) Cytotoxicity was assessed by a 6-hour ^{51}Cr-release assay at E:T ratio 9:1 and 1:1. Technical triplicates are depicted in the graph and the values are a representatives of at least two independent experiments.

Table S1. Peptide-HLA complexes and selected MAGE-specific T cell clones							
					Recognition pattern identified T cell clones		
Target peptide	HLA-restriction	Target gene	Eluted from	NetMHC[A]	Td gene[B]	Potent & safe[C]	Final[D]
ALIEVGPDHFC	A*02:01	*MAGE-C2*	OvaL10	7206	-	-	
ALKDVEERV	A*02:01	*MAGE-C2*	-	470	1	-	
ALKLKVAEL	B*08:01	*MAGE-A9*	RPMI8226 +B8	71	-	-	
ALREEEEGV	A*02:01	*MAGE-A1*	U266, C4-2B4, OvaL11	825	1	-	
EADPTGHSY	A*01:01	*MAGE-A1*	-	42	2	-	
EVDPIGHLY	A*01:01/<br>B*35:01	*MAGE-A3*	-<br>-	11<br>297	7<br>6	-<br>3	-<br>1(2H9)
FPSLREAAL	B*07:02	*MAGE-A1*	U266 and OvaL11	9	2	-	
FVYGEPREL	A*02:01	*MAGE-C2*	U266	512	3	-	
GLLGDNQIMPK	A*03:01	*MAGE-A1/A3/A6*	U266, RPMI8226 +A3	206	3	-	
GVYAGREHFV	A*02:01	*MAGE-C2*	U266	975	2	-	
GVYDGREHTV	A*02:01	*MAGE-A4*	U266, UM9 +A2, OvaL10	1752	1	-	
IMPKAGLLII	A*24:02	*MAGE-A3*	OvaL1	413	-	-	
IVLGVILTK	A*03:01	*MAGE-A9*	RPMI8226 +A3	27	-	-	
KIWEELSVLEV	A*02:01	*MAGE-A3/A6*	U266	87	-	-	
KVLEFLAKL	A*02:01	*MAGE-C2*	U266	25	3	-	
KVLEYVIKV	A*02:01	*MAGE-A1*	U266	7	18	3	1 (4F7)
LTQDLVQEKYLEY	A*01:01	*MAGE-A1*	RPMI8226 +A1	126	1	1	1 (6G4)
LVFGIELMEV	A*02:01	*MAGE-A3*	-	41	6	-	
RCFPVIFGK	A*03:01	*MAGE-A4*	U266, UM9 +A3	217	-	-	
RPADLTRVIM	B*07:02	*MAGE-A11*	Oval10	9	-	-	
RVRFFFPSL	B*07:02	*MAGE-A1*	U266	75	61	3	1 (3G2)
RVRFFFPSLR	A*03:01	*MAGE-A1*	Prediction	46	-	-	
RVRIAYPSL	B*07:02	*MAGE-A4*	U266	53	-	-	
RVRIAYPSLR	A*03:01	*MAGE-A4*	U266	120	7	-	
SLFRAVITK	A*03:01	*MAGE-A1*	U266	14	39	2	1 (3H4)
SMLGDGHSMPK	A*03:01	*MAGE-A9*	RPMI8226 +A3	68	-	-	
SVMGVYVGK	A*03:01	*MAGE-A9*	RPMI8226+A3	29	-	-	
TLDEKVAEL	A*02:01	*MAGE-C2*	U266	19	2	-	
TQDLVQEKY	A*01:01	*MAGE-A1/B1/B4*	RPMI8226 +A1	562	-	-	
VAELVHFLL	A*24:02	*MAGE-A3*	-	7689	1	-	
VIWEVLNAV	A*02:01	*MAGE-C2*	-	11	5	-	
VLGEEQEGV	A*02:01	*MAGE-A9*	RPMI8226 +A2	167	-	-	
VRFFFPSL	C*07:01/<br>C*07:02	*MAGE-A1*	Prediction/<br>U266	8807<br>5858	2<br>13	-<br>1	-<br>1(10C1)
YPSLREAAL	B*07:02	*MAGE-A4*	U266, UM9	7	-	-	
YVGKEHMF	A*24:02	*MAGE-A9*	RPMI8226 +A24	3852	-	-	
YVGKEHMFY	A*01:01	*MAGE-A9*	RPMI8226 +A1	341	1	1	1 (2D8)

MAGE specific peptides were identified form multiple myeloma cell lines U266, UM9 and RPMI8226; prostate cancer cell line C4-2B4, and primary ovarium carcinoma samples OvaL1, OVal10, and Oval11.

[A]Predicted NetMHC 4.0 affinity (nM)

[B]T cell clones produce IFN-γ after stimulation with MAGE-gene transduced Raji cell.

[C]T cell clones that show potent and strict recognition against Tumor cell line panels, EBV-LCL panel and MAGE-A panel.

[D]The final selection of most promising T cell clones for TCR gene therapy

Table S2. HLA-typing of EBV-LCLs used in EBV-LCL screenings

EBV-LCL	HLA-A	HLA-B	HLA-C
55	01 – 68	08 – 53	04 - 07
1854	02:01 - 30:02	15:01 - 39:01	03:03 - 12:03
2065	01:01 - 02:01	37:01 - 39:01	06:02 - 07:02
2631	02:01 - 03:01	44:02 - 57:01	06:02 - 07:04
3281	01:01 - 32:01	08:01 - 45:01	06:02 - 07:01
3480	26:01 - 01:01	38:01 - 18:01	12:03 - 07:01
3516	03:01 - 26:01	07:02 - 14:01	07:02 - 08:02
3829	01:01 - 68:01	44:02	05:01 - 07:04
4084	03:01- 30:01	07:02 - 38:01	07:02 - 12:03
4582	01 - 30:01	08:01 -40:01	03:04 - 07:01
4803	03:01 - 33:01	07:02 - 14:02	07:02 - 08:02
4991	26:01 - 31:01	14:01 - 49:01	07:01 - 08:02
5171	02:01 - 66:01	40:01 - 41:02	03:04 - 17
5283	24:02 - 66:01	14:02 - 39:06	07:02 - 08:02
5524	02:01 - 31:01	15:01 - 15:17	03:04 - 07:01
5439	03:01 - 25:01	15:17 - 18:01	07:01 - 12:03
5696	02:05	58:01	unknown
5702	32:01 - 68:01	35:03 - 52:01	12:02 - 12:03
5769	02:01 - 68:01	35:03 - 37:01	04:01 - 06:02
5857	30:04 - 68:02	38:01 - 55:01	03:03 - 12:03
5866	02:01 - 11:01	35:01 - 51:01	04:01 - 14:02
5989	11:01 -68:01	38:01 - 39:01	07:02 - 12:03
6023	03:01 - 11:01	40:02 - 56:01	01:02 - 02:02
6268	02:01 - 24:02	35:02 - 44:02	04:01 - 05:01
6316	29:02 - 30:01	13:02 - 44:03	06:02 - 16:01
6461	02:01	40:02	02:02
6463	02:01	57:01	06:02
6945	03:01 - 25:01	18:01 - 35:01	04:01 - 12:03
6978	02:01 - 02:05	15:01 - 45:01	01:02 - 06:02
7007	02:05 - 29:02	27:05 - 44:03	01:02 - 16:01
7159	02:01	13:02 - 44:02	05:01 - 06:02
7394	01:01 - 32:01	35:08	04:01
7590	24:02 - 31:01	07:02 - 35:08	04:01 - 07:02
7613	01:01 - 24:02	15:01 - 37:01	03:03 - 06:02
7929	01:01	35:02- 52:01	04:01 - 12:02
8419	02:01 - 01:01	50:01 - 07:02	07:02 - 06:02
8425	23:01 - 02:01	41:01 - 40:01	17:01 - 03:04
8428	11:01 - 01:01	57:01 - 35:01	06:02 - 04:01
8463	11:01 - 01:01	51:01 - 50:01	15:02 - 06:02
8466	23:01	51:01 - 41:01	17:01 - 15:02
9210	02:01	15:01 - 51:01	03:03 - 15:02
9271	33:01 - 66:01	58:01 - 58:02	03:02- 06:02
9619	01:01 - 33:03	44:03 - 51:01	07:06 - 14:02
9877	01:01 - 23:01	08:01 - 41:02	07:01 - 17:03
9779	02:01 - 03:01	08:01 - 50:01	06:02 - 07:01
10120	02:01 - 24:02	07:02 - 40:02	02:02 - 07:02
10299	02:01 - 11:01	44:05 - 51:01	02:02 - 14:02
12425	23:01 - 36:01	15:03 - 53:01	02:10 - 04:01

Table S3. Primers included in the RT-qPCR experiments		
**Gene**	**Forward primer**	**Reverse primer**
MAGE-A1	GAGTCCTTGTTCCGAGCAGT	GGCTCCCTGGCTCGATATTT
MAGE-A3/A6	CCTGAGCAACGAGCGACG	TCAGAACCTTGCCTCCTCACC
MAGE-A9	GATCCTGCGCACTACGAGTT	ATGGGTAGCAGATGGGCTCT
GUSB	ACTGAACAGTCACCGACGAG	GGAACGCTGCACTTTTTGGT
PSMB4	GTTTCCGCAACATCTCTCGC	CATCAATCACCATCTGGCCG
VPS29	TGAGAGGAGACTTCGATGAGAATC	TCTGCAACAGGGCTAAGCTG
