# Supplementary material for: A library of cancer testis specific T cell receptors for T cell receptor gene therapy
Source: Mol Ther Oncolytics. 2022 Dec 2;28:1–14. doi: 10.1016/j.omto.2022.11.007 (PMC9792401; doi:10.1016/j.omto.2022.11.007)
Supplement: Document S2. Article plus supplemental information [file mmc2.pdf]

Molecular Therapy
Oncolytics
Original Article

A library of cancer testis specific T cell receptors for T cell receptor gene therapy

Marije A.J. de Rooij,[1] Dennis F.G. Remst,[1] Dirk M. van der Steen,[1] Anne K. Wouters,[1] Renate S. Hagedoorn,[1] Michel G.D. Kester,[1] Miranda H. Meeuwsen,[1] Tassilo L.A. Wachsmann,[1] Arnoud H. de Ru,[2] Peter A. van Veelen,[2] Els M.E. Verdegaal,[3] J.H. Frederik Falkenburg,[1] and Mirjam H.M. Heemskerk[1]

[1]Department of Hematology, Leiden University Medical Center, Albinusdreef 2, 2333 ZA Leiden, the Netherlands; [2]Center for Proteomics and Metabolics, Leiden University Medical Center, 2333 ZA Leiden, the Netherlands; [3]Department of Medical Oncology, Oncode Institute, Leiden University Medical Center, 2333 ZA Leiden, the Netherlands

To increase the number of cancer patients that can be treated with T cell receptor (TCR) gene therapy, we aimed to identify a set of high-affinity cancer-specific TCRs targeting different melanoma-associated antigens (MAGEs). In this study, peptides derived from *MAGE* genes with tumor-specific expression pattern were identified by human leukocyte antigen (HLA) peptidomics. Next, peptide-HLA tetramers were generated, and used to sort MAGE-specific CD8$^+$ T cell clones from the allogeneic (allo) HLA repertoire of healthy donors. To evaluate the clinical potential, most potent TCRs were sequenced, transferred into peripheral blood-derived CD8$^+$ T cells, and tested for antitumor efficacy. In total we identified, seven MAGE-specific TCRs that effectively target MAGE-A1, MAGE-A3, MAGE-A6, and MAGE-A9 in the context of HLA-A*01:01, -A*02:01, -A*03:01, -B*07:02, -B*35:01, or -C*07:02. TCR gene transfer into CD8$^+$ T cells resulted in efficient reactivity against a variety of different tumor types, while no cross-reactivity was detected. In addition, major *in vivo* antitumor effects of MAGE-A1 specific TCR engineered CD8$^+$ T cells were observed in the orthotopic xenograft model for established multiple myeloma. The identification of seven MAGE-specific TCRs expands the pool of cancer patients eligible for TCR gene therapy and increases possibilities for personalized TCR gene therapy.

INTRODUCTION

Melanoma-associated antigen (MAGE) genes are potential targets for T cell-based immunotherapy since they are highly expressed in multiple different tumor types as demonstrated by The Cancer Genome Atlas (TCGA) Research network (https://www.cancer.gov/tcga). Expression in normal tissue is in general restricted to the immune privileged testis and placenta, which makes MAGE antigens ideal targets for T cells.[1–4] T cells with high-affinity T cell receptors (TCRs) targeting self-antigens, such as *MAGE* genes, are deleted in the self-repertoire during negative selection in the thymus.[5,6] Therefore, MAGE-specific T cells present in the self-repertoire may display only limited antitumor efficacy, as illustrated by the limited antitumor responses in patients after vaccination aiming at harnessing MAGE-specific T cells.[7,8] To circumvent negative selection of high-affinity TCRs in the thymus, high-affinity TCRs specific for self-antigens can be identified from the T cell repertoire of human leukocyte antigen (HLA)-mismatched individuals, also known as allogeneic (allo) HLA.[5,6,9] Previous studies revealed that self-antigens can provoke strong immune responses when presented in the context of allo-HLA molecules.[10] Recently, various potent TCRs have been identified from the allo-HLA repertoire, of which several are in the process of clinical development.[10–13] Despite the promise for potent antitumor reactivity, the use of high-affinity TCRs for TCR gene therapy also comes with a toxicity risk. Two clinical trials targeting an MAGE-A3 peptide had to be terminated because unexpected off-target toxicity resulted in deaths of patients.[14,15] In the first clinical trial, an affinity-optimized TCR was used, which in addition to the targeted peptide KVAELVHFL peptide from MAGE-A3, also recognized the KMAELVHFL peptide of MAGE-A12. The expression of MAGE-A12 in the brain most likely resulted in death of two patients. In a second study, an MAGE-A3 targeting TCR recognizing EVDPIGHLY peptide, additionally recognized the ESDPIVAQY peptide derived from the titin protein, which led to cardiotoxicity and a fatal outcome in two patients. These observations underline the importance of thorough safety screenings during the selection process of tumor-specific TCRs to exclude TCR cross-reactivity with homologous peptides.[14–16]

In this study, we aimed to identify potent MAGE-specific TCRs. All MAGE-specific TCRs together can form an MAGE-TCR-library, which could be used for personalized TCR gene therapy, based on HLA typing of the patient and gene expression of the tumor. The TCR constructs can be generated in advance and used as an "off-the-shelf" TCR library that is readily available when required for transduction (Td) of the patient's isolated CD8$^+$ T cells. As opposed to neo-antigen specific TCR therapy, this will reduce production time,

Received 8 June 2022; accepted 29 November 2022;
https://doi.org/10.1016/j.omto.2022.11.007.

Correspondence: Marije A.J. de Rooij, Department of Hematology, Leiden University Medical Center, Albinusdreef 2, 2333 ZA Leiden, the Netherlands.
E-mail: m.a.j.de_rooij@lumc.nl
Correspondence: Mirjam H.M. Heemskerk, Department of Hematology, Leiden University Medical Center, Albinusdreef 2, 2333 ZA Leiden, the Netherlands.
E-mail: m.h.m.heemskerk@lumc.nl

This is an open access article under the CC BY license (http://creativecommons.org/licenses/by/4.0/).

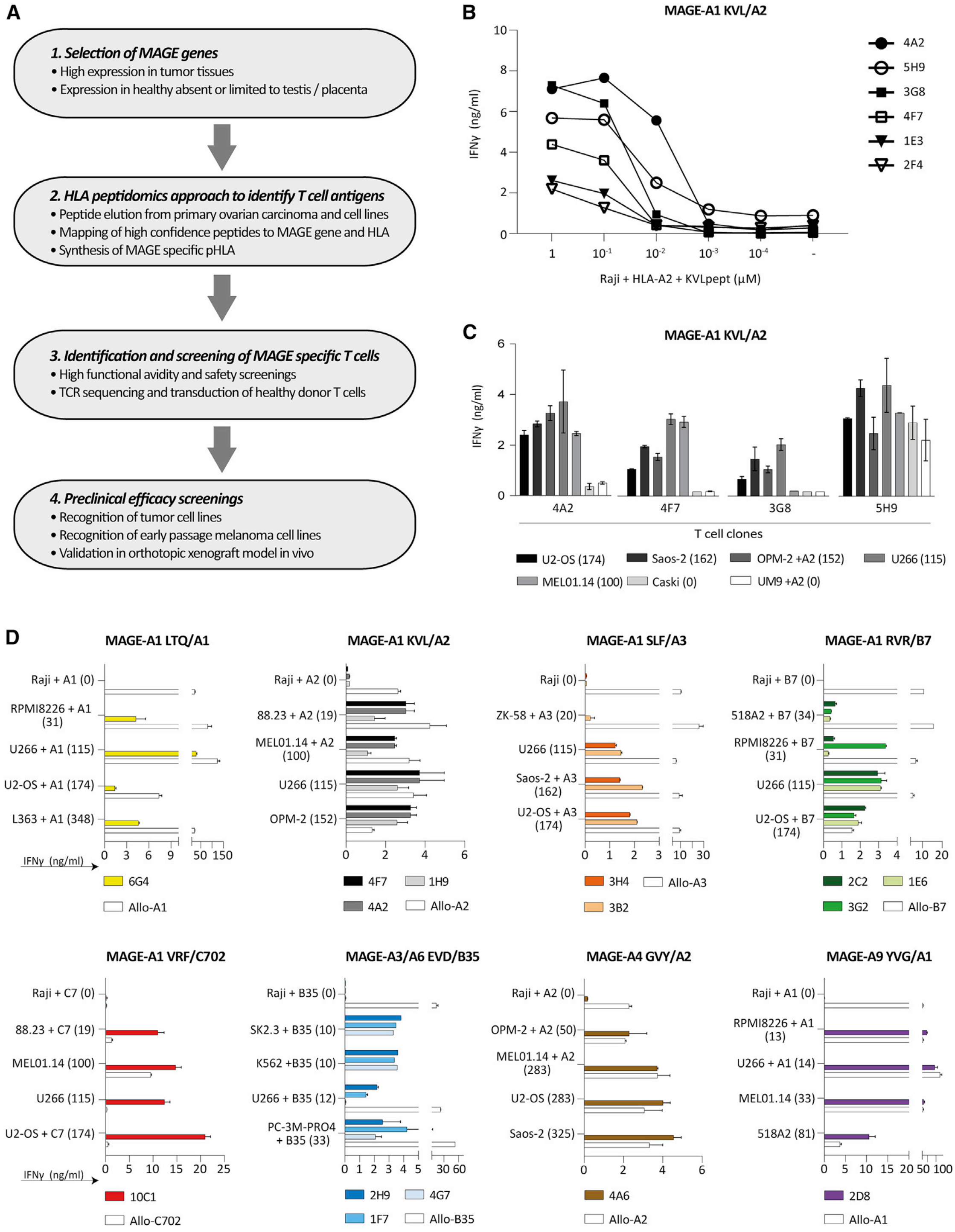

Figure 1. Selection of MAGE-reactive T cells

(A) Flowchart describing the four steps for isolation of MAGE-reactive T cell clones. T cell clones were selected based on peptide titration experiment and specific tumor cell line recognition. (B) Six of 18 T cell clones recognizing MAGE-A1 KVLEYVIKV in HLA-A*02:01 (MAGE-A1 KVL/A2) are depicted in this graph that were overnight co-cultured with KVL peptide loaded Raji cells. These six T cell clones are a representative for T cell clone selection process of all 187 MAGE-specific T cell clones. (C and D) T cell clones were stimulated with tumor cell lines of different origin, including multiple myeloma (RPMI8226, U266, L363, OPM-2, UM9), osteosarcoma (U2-OS, ZK-58, Saos-2), melanoma (88.23, MEL01.14, SK2.3, 518A2, OPM-2), chronic myeloid leukemia k562, cervix carcinoma Caski, and prostate carcinoma cell line PC-3M-PRO4. T cell reactivity

(legend continued on next page)

costs, and workload, since unique tumor-reactive TCRs do not have to be identified on a per-patient-basis. Moreover, expanding the TCR library makes it possible to treat individual patients with combination therapy targeting multiple genes. Targeting multiple antigens in a single therapy is expected to enhance the efficacy of TCR gene therapy by reducing the likelihood of tumor escape.

To identify MAGE-specific TCRs, we used bioinformatic tools to select the most promising *MAGE* genes to target, and from these genes the naturally processed and presented HLA class-I peptides were identified. Peptide-HLA (pHLA) tetramers were used to isolate MAGE-antigen-specific T cells from the allo-HLA repertoire of healthy individuals. In total, we identified seven TCRs with an effective and specific recognition profile for MAGE-A1, MAGE-A3, MAGE-A6, and MAGE-A9 in the context of HLA-A*01:01, -A*02:01, -A*03:01, -B*07:02, -B*35:01, or -C*07:02. These identified TCRs allow TCR gene therapy for an increased number of cancer patients, and development of combination TCR gene therapy.

RESULTS

Selection of promising *MAGE* genes and candidate peptides

We developed a bioinformatics pipeline combined with an immunopeptidomics approach to identify antigens derived from the MAGE family suitable for TCR-T cell therapy (Figure 1A). In this pipeline, those CT genes described in the CT database (http://www.cta.lncc.br/) with high expression (RNA sequencing V2 (log) > 6) in TCGA database, and in addition expressed in a substantial percentage (>10%) of certain tumor types were included. Subsequently, only those CT genes with restricted expression in testis and/or placenta demonstrated by the Genotype-Tissue Expression (GTEx), and Human Protein Atlas (HPA) were selected. Using these publicly available databases, we identified *MAGE-A1*, *MAGE-A3*, *MAGE-A4*, *MAGE-A6*, *MAGE-A9*, *MAGE-A11 MAGE-C1,* and *MAGE-C2* to have an attractive expression profile (Figure 1A step 1). These genes are highly expressed and prevalent in tumor tissues, while their expression in healthy tissues is absent or limited to testis or placenta. We then proceeded to identify putative T cell antigens using an immunopeptidomics-based approached (Figure 1A step 2). Immunopeptidomics was performed on eight resected ovarian cancers, three multiple myeloma (MM) cell lines (U266, RPMI8226, and UM9), and one prostate carcinoma cell line (C4-2B4) (Table S1). MAGE peptides with an Ion Score $\geq$30 were selected, and validated by comparing mass spectra of eluted peptides and synthetic peptides (example in Figure S1). Eluted peptides were matched to HLA alleles by combining predicted HLA binding (netMHC4.0) with the HLA typing of the material from which the peptides originated. Peptides binding in prevalent HLA alleles (HLA-A*01:01, -A*02:01, -A*03:01, A*11:01, -A*24:02, -B*07:02, -B*08:01, -B*35:01, -C*07:01, and -C*07:02) were selected. To avoid off-target toxicity, peptides with an identical peptide sequence in any other human gene than the selected *MAGE* genes as determined by BLAST search (blast.ncbi.nlm.nih.gov) were excluded. By this method, 29 different MAGE peptides were identified, of which most have been described before. Two new peptides derived from MAGE-A9, YVGKEHMF binding in HLA-A*24:02 and SMLGDGHSMPK binding in HLA-A*03:01, were not described previously according to the iedb.org database (Figure S1). In addition to the set of peptides identified in our peptidomics approach, seven peptide-HLA (pHLA) combinations were included based on literature; furthermore, two pHLA combinations were included based on predicted binding by netMHC4.0 (Table S1). For all peptides, pHLA monomers were successfully refolded, demonstrating stable binding to the respective HLA molecules (data not shown). Overall, peptides derived from MAGE-A1, MAGE-A3, MAGE-A4, MAGE-A6, MAGE-A9, MAGE-A11, and MAGE-C2 were identified and selected. Most peptides originated from MAGE-A1 (11 peptides), followed by MAGE-A9 (seven peptides) and MAGE-A3 (six peptides). We did not identify peptides from MAGE-C1. In total, 38 different peptide-HLA combinations were used to generate monomers of which PE-labeled pHLA tetramers were constructed and used for MAGE-specific T-cell isolation.

Isolation of MAGE-specific T cells from the allo-HLA repertoire

To identify MAGE-specific T cells, peripheral blood mononuclear cells (PBMCs) (5–10 $\times$ 10^9 cells) from in total 54 different HLA-typed healthy donors were separately incubated with a pool of phycoerythrin (PE)-labeled pHLA tetramers. pHLA tetramers were only included when donors were negative for the respective target HLA allele. In total, 22,344 CD8^{pos}tetramer^{pos} cells (ranging between 10 and 1,920 per donor) were single-cell sorted, of which on average 60% clonally expanded. Initially, all isolated T cell clones were screened for peptide-specific recognition using peptide-pool loaded Raji cells that were Td with HLA alleles of interest (except for HLA-A*03:01 that is naturally expressed on Raji cells). T cell clones that produced interferon (IFN)-γ upon co-culture with peptide-pool loaded HLA-Td Raji cells, but not unloaded HLA-Td Raji, were selected. To evaluate the recognition of naturally processed and presented peptides by the selected T cell

was demonstrated by IFN-γ production, as measured by ELISA, after an overnight co-culture. *MAGE*-gene expression levels, measured by qPCR, are depicted between brackets as percentage relative to HKGs. Targeted HLA was naturally expressed or transduced (+) into the target cell. (C) Four of 18 MAGE-A1 KVL/A2 recognizing T cell clones are depicted as representatives. T cell clones 4A2 and 4F7 strictly recognize the MAGE-expressing tumor cell lines efficiently and therefore selected for further analysis. (D) Results of all selected T cell clones reactive against MAGE-A1 LTQDLVQEKYLEY in HLA-A*01:01 (MAGE-A1 LTQ/A1), MAGE-A1 KVL/A2, MAGE-A1 SLFRAVITK in HLA-A*03:01 (MAGE-A1 SLF/A3), MAGE-A1 RVRFFFPSL in HLA-B*07:02 (MAGE-A1 RVR/B7), MAGE-A1 VRFFFPSL in HLA-C*07:02 (MAGE-A1 VRF/C7), MAGE-A3/A6 EVDPIGHLY/EVDPIGHVY in HLA-B*35:01 (MAGE-A3/A6 EVD/B35), and MAGE-A1 YVGKEHMFY in HLA-A*01:01 (MAGE-A9 YVG/A1). The Burkitt lymphoma Raji, negative for all MAGE antigens, was included as negative control. To confirm proper HLA expression and recognition capacity of the targets allo-HLA reactive T cell clones (white bars) were included for each HLA specificity. Values and error bars represent mean and standard deviations of technical duplicates. Experiments are representative of at least two independent experiments.

clones, HLA-Td Raji cells were additionally Td with *MAGE* genes. T cell clones (n = 187) that specifically produced IFN-γ upon stimulation with MAGE-Td Raji were selected for further screening (Table S1).

Tumor reactivity of MAGE-specific T cell clones against MAGE-positive tumor cell lines

To allow selection of T cell clones with the highest functional avidity, peptide titration experiments followed by IFN-γ ELISA was performed with all 187 selected T cell clones (example in Figure 1B). Subsequently, the T cell clones were screened against *MAGE* gene-positive and -negative tumor cell lines that naturally expressed or were Td with HLA alleles of interest. The *MAGE*-gene expression levels of included tumor cell lines were determined by quantitative reverse transcription polymerase chain reaction (qPCR) (Figure S2). T cell clones with a high functional avidity that potently recognize MAGE-positive tumor cells lines but do not recognize negative tumor cell lines, like clones 4A2 and 4F7, were selected (Figure 1C). In total, 15 T cell clones specific for peptides from MAGE-A1, MAGE-A3, MAGE-A4, or MAGE-A9 in the context of HLA-A*01:01, HLA-A*02:01, HLA-A*03:01, HLA-B*07:02, HLA-B*35:01, or HLA-C*07:02 were selected for further investigation (Figure 1D).

Selection of high-affinity TCRs with a safe recognition profile

Next to specific recognition, TCRs suitable for clinical translation need to have a safe recognition profile. To unravel potential major cross-reactivities with other HLA molecules, MAGE-specific T cell clones were subjected to a safety screening using an EBV-LCL panel. The panel consists of EBV-LCLs, covering all HLA alleles that have a prevalence of >1% in the Caucasian population (Table S2). One MAGE clone; 4A2 (MAGE-A1 KVL/A2) was excluded based on the recognition of two EBV-LCLs, as determined by IFN-γ production upon overnight co-culture (Figure S3). The exact peptide-HLA complex that was recognized by clone 4A2 (MAGE-A1 KVL/A2) could not be determined, since the recognized EBV-LCLs did not express a shared HLA allele. Clone 3G2 (MAGE-A1 RVR/B7) recognized HLA-A*68:02-positive EBV-LCLs. However, since the HLA allele frequency of expression of HLA-A*68:02 is low (<1%) in the Caucasian population, this clone was not excluded from further screenings.

Furthermore, the MAGE-specific TCRs could potentially be cross-reactive with peptides derived from highly homologous MAGE family gene members that have an unsafe expression profile.[17,18] To exclude this, the 14 residual MAGE clones were tested using Raji cells separately Td with all MAGE-A family members and relevant HLA alleles to identify clones recognizing a peptide present in one of the non-selected MAGE-A genes. Most MAGE clones, except for three, only recognized their MAGE-target gene as determined by IFN-γ production upon overnight co-culture (Figure 2A). Clones 2H9 and 4G7 (MAGE-A3 EVD/B35) were additionally reactive against MAGE-A6. Since MAGE-A6 was previously selected as a safe MAGE gene, these two clones were not excluded from further screening. However, to investigate if recognition of MAGE-A6 resulted from the sequence homology between MAGE-A3 and MAGE-A6, the EVD peptide from MAGE-A6 (EVDPIGHVY) as well as target EVDPIGHLY peptide from MAGE-A3 were loaded on Raji cells. Recognition of MAGE-A6^{EVD} as well as MAGE-A3^{EVD} peptide loaded Raji cells demonstrated that the high peptide similarity resulted in recognition of both *MAGE* genes (Figure 2B). The 4A6 (MAGE-A4 GVY/A2) clone reactive against GVYDGREHTV peptide also recognized MAGE-A8. Since MAGE-A8 is lowly expressed in cerebellum, thyroid gland, and urinary bladder, according to the online databases GTEx and HPA, this T cell clone was therefore excluded.

Since cross-reactivities may occur from peptides beyond the MAGE gene family, the 13 residual T cell clones were co-cultured with a large cell panel of *MAGE*-gene negative tumor cell lines and healthy cell subsets from multiple tissue origins, all expressing the required HLA-restriction alleles. None of the T cell clones produced IFN-γ upon stimulation with these *MAGE*-gene negative tumor cell lines and healthy cell subsets, whereas Raji Td with HLA and *MAGE*-target gene were recognized (Figure 3). Based on these safety screenings, we concluded that the 13 selected MAGE clones, specific for seven different MAGE peptides, have a safe reactivity profile. For some specificities, multiple promising T cell clones were identified; for these specificities the most potent candidates were selected for TCR gene therapy (Tables 1 and S1).

Gene transfer of MAGE-specific TCR into healthy donor T cells results in potent and specific tumor recognition *in vitro*

To study the potential for clinical application in TCR gene therapy, the TCRs of the seven selected T cell clones were sequenced and retrovirally transferred into healthy donor CD8$^+$ T cells. All TCRs were combined with cysteine-modified murine TCR-α and -β constant domains in the retroviral vector to prevent mispairing with the endogenous TCRs. Tetramer and murine TCR-β staining confirmed TCR expression in Td CD8$^+$ T cells (Figure 4A). Tetramer staining of the 10C1 (MAGE-A1 VRF/C7) and 2H9 (MAGE-A3 EVD/B35) TCR Td T cells (TCR-T cells) had lower gMFI compared with other selected TCRs. Nevertheless, all TCR-T cells demonstrated effective cytotoxicity against tumor cell lines expressing the relevant *MAGE* genes, while target gene negative cells were not killed (Figure 4B). CMV TCR-T cells and allo-HLA reactive clones were included to demonstrate TCR-specific killing, and killing sensitivity of tumor cell lines, respectively (Figure S4). These results demonstrated that the seven identified TCRs specific for MAGE-A1, MAGE-A3, MAGE-A6, or MAGE-A9 in the context of HLA-A*01:01, -A*02:01, -A*03:01, -B*07:02, -B*35:01, or -C*07:02 were promising TCRs for further investigation of antitumor reactivity.

TCR transfer reveals effective cytokine production and killing of early-passage melanoma samples

To obtain further insight into the potential clinical effectivity of the TCRs, antitumor reactivity of TCR-T cells was analyzed using early-passage melanoma cell lines (passage ≤10). Expression of different *MAGE* genes in these cell lines was measured by qPCR (Figure S5), and was demonstrated to be comparable to levels described in literature.[18] For each TCR, one or two early-passage melanoma cell

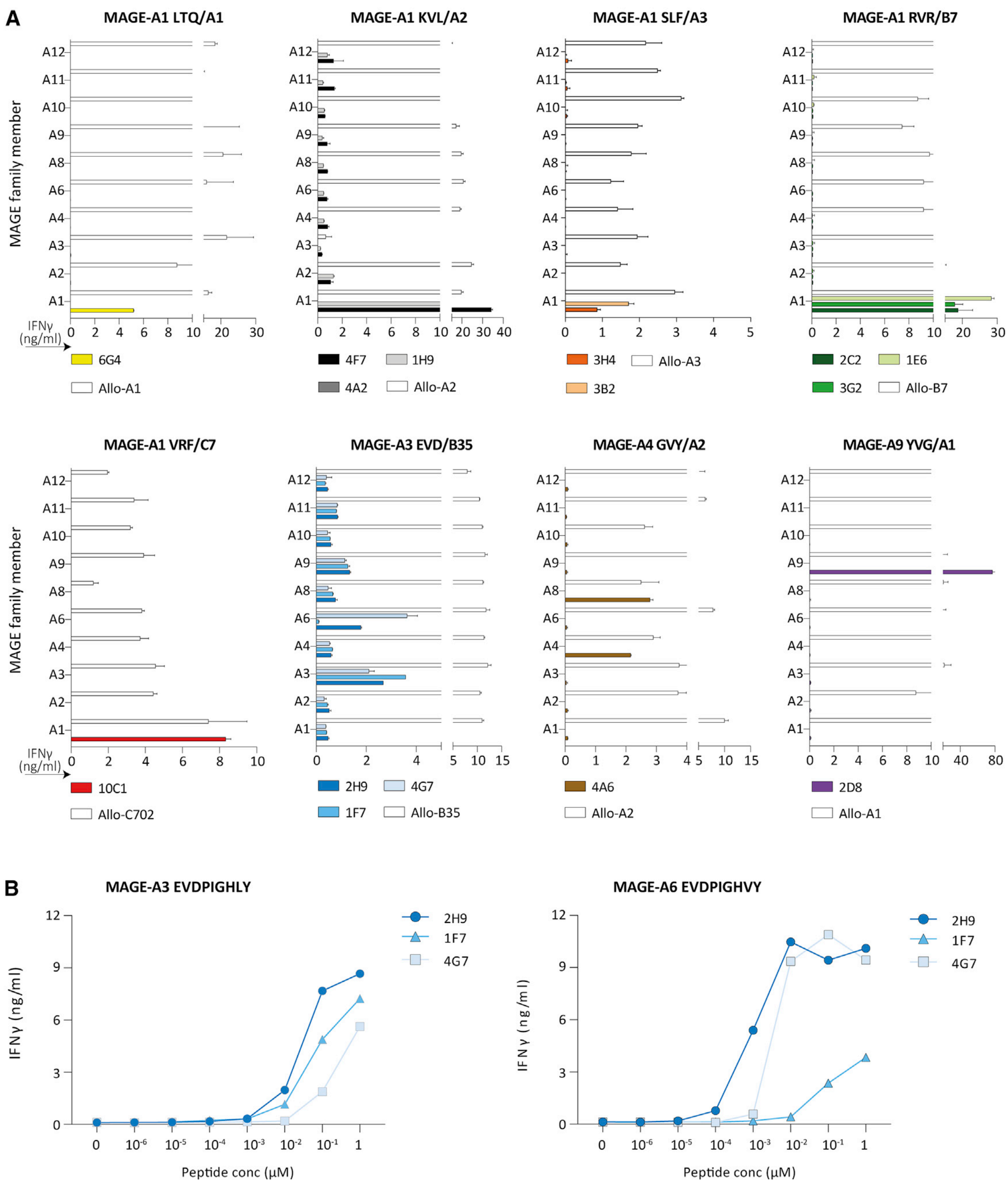

Figure 2. Recognition of specific MAGE-A family members by our selected T cell clones

(A) T cell clones were stimulated with Raji transduced with the different MAGE-A family members, including MAGE-A1 (A1), MAGE-A2 (A2), MAGE-A3 (A3), MAGE-A4 (A4), MAGE-A6 (A6), MAGE-A8 (A8), MAGE-A9 (A9), MAGE-A10 (A10), MAGE-A11 (A11), and MAGE-A12 (A12). Cytokine production was measured by IFN-γ ELISA after an overnight co-culture. In all experiments, an allo-HLA reactive T-cell clone (white bars) was included to confirm proper HLA expression and stimulatory capacity of the targets. Values and error bars represent means and standard deviations of technical duplicates. (B) In peptide titration experiments the three selected MAGE-A3/A6 EVD/B35 T cell clones were separately stimulated with MAGE-A3^{EVD} or MAGE-A6^{EVD} peptide loaded on Raji cells. T cell reactivity was determined by IFN-γ production, as measured by ELISA.

lines expressing both the target gene and HLA of interest, were included. Antitumor reactivity of the TCR-T cells was evaluated by IFN-γ production after an overnight co-culture with early-passage melanoma cell lines pre-treated with IFN-γ and in cytotoxicity experiments. CMV TCR-T cells and allo-HLA reactive clones were included to demonstrate TCR-specific killing, and killing sensitivity of the early-passage melanoma cell lines, respectively (Figure S4). Six TCR-T cell products produced high amounts of IFN-γ and

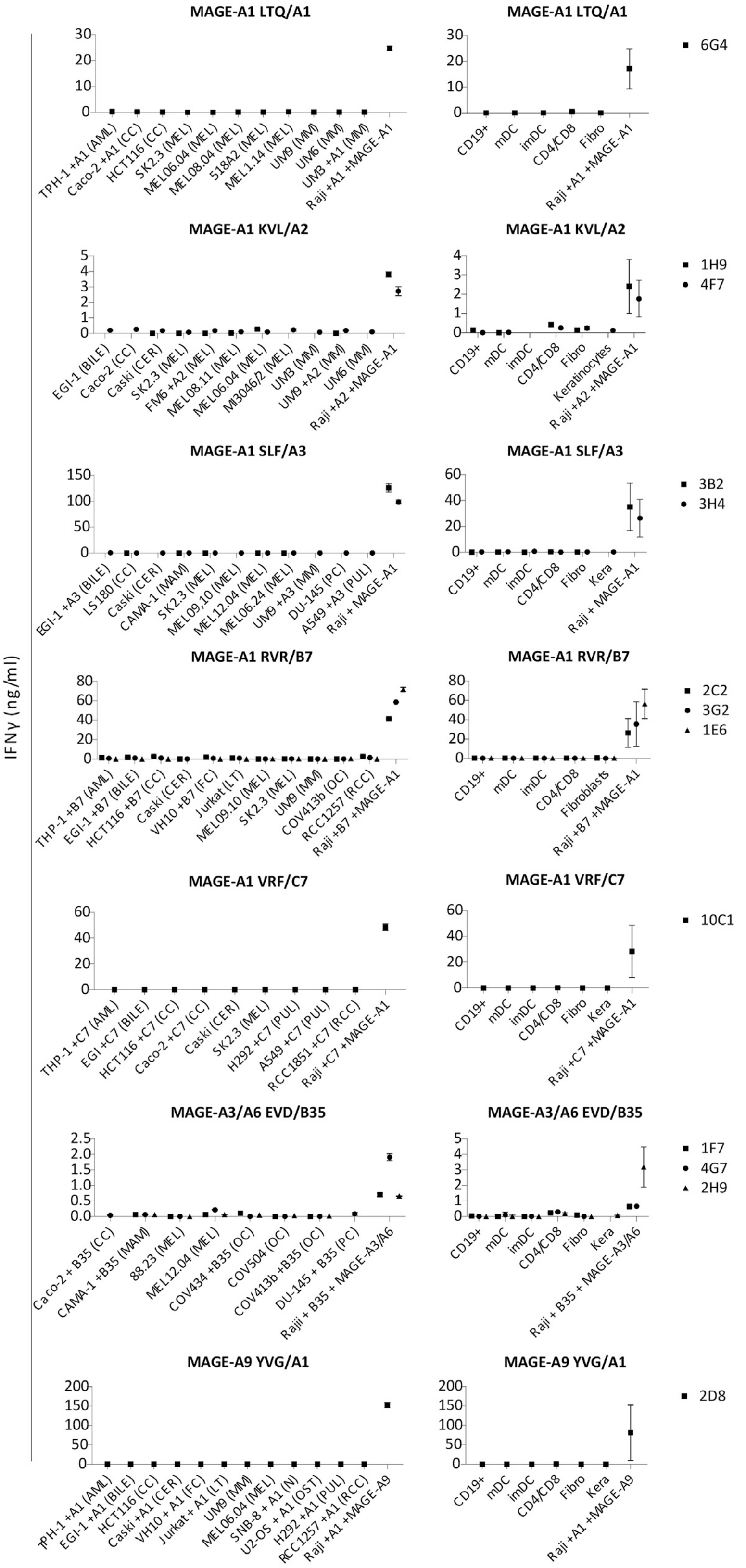

Figure 3. Cross-reactivity against a peptide in the context of the targeted HLA

Cross-reactivity was determined by an overnight co-culture of T cells with *MAGE*-gene negative tumor cell lines (left side) and healthy subsets (right side) from different tissue origin. The cell lines included originated from acute myeloid leukemia (AML), bile duct carcinoma (BILE), colon carcinoma (CC), cervical carcinoma (CER), T cell leukemia (LT), melanoma (MEL), multiple myeloma (MM), ovarian carcinoma (OC), prostate carcinoma (PC), and pulmonary carcinoma (PUL) and fibroblast cell lines (FC). The healthy subsets included were CD19-positive cells ($CD19^+$), immature dendritic cells (imDC), mature dendritic cells (mDC), activated T cells (CD4/CD8), fibroblasts (Fibro), and keratinocytes (Kera). Each dot in the healthy subset panel represents the average of at least two of these cell subsets derived from different healthy donors. All included targets expressed the MAGE-A genes of interest <1% relative to HKGs and naturally expressed the HLA of interest or were HLA-Td (+HLA). IFN-γ production was measured by ELISA after an overnight co-culture assay. Proper HLA presentation and recognition capacity of the target cells was confirmed by allo-HLA reactive T cell clones (data not shown). Raji transduced or naturally expressing the HLA and *MAGE* gene of interest was included as a positive control. Error bars depict standard deviations.

Table 1. TCRs included in the TCR library

Name	Peptide	Gene	HLA-restriction	TCR target abbreviations
6G4	LTQDLVQEKYLEY	*MAGE-A1*	HLA-A*01:01	MAGE-A1 LTQ/A1
4F7	KVLEYVIKV	*MAGE-A1*	HLA-A*02:01	MAGE-A1 KVL/A2
3H4	SLFRAVITK	*MAGE-A1*	HLA-A*03:01	MAGE-A1 SLF/A3
3G2	RVRFFFPSL	*MAGE-A1*	HLA-B*07:02	MAGE-A1 RVR/B7
10C1	VRFFFPSL	*MAGE-A1*	HLA-C*07:02	MAGE-A1 VRF/C7
2H9	EVDPIGHLY/ EVDPIGHVY	*MAGE-A3/A6*	HLA-B*35:01	MAGE-A3/A6 EVD/B35
2D8	YVGKEHMFY	*MAGE-A9*	HLA-A*01:01	MAGE-A9 YVG/A1

effectively lysed all MAGE-positive early-passage melanoma cell lines upon stimulation (Figures 5A and 5B).

One T cell clone, clone 6G4 (MAGE-A1 LTQ/A1), strongly recognized all HLA-A*01:01-positive MM cell lines but produced limited amounts of IFN-γ upon co-culture with early-passage melanoma cell lines and other tumor cell lines of non-hematopoietic origin (Figure S6). Based on this potent anti-MM reactivity TCR 6G4 was selected as a promising TCR for TCR gene therapy of MM. In conclusion, we identified seven promising MAGE-specific TCRs, of which the majority potently lysed short-term cultured melanoma and all TCR-T cells lysed MM cell lines, implicating broad applicability for TCR gene therapy.

Potent antitumor reactivity of MAGE-A1 TCRs *in vivo*

In vivo efficacy of three of the identified TCRs, namely, 4F7 (MAGE-A1 KVL/A2), 3H4 (MAGE-A1 SLF/A3), and 10C1 (MAGE-A1 VRF/C7), were determined in an orthotopic xenograft model for established MM.[12] To this end, MM cell line U266 was used, which endogenously expresses HLA-A*02:01, HLA-A*03:01, HLA-C*07:02, and MAGE-A1. NSG mice were intravenously injected with U266 cells, 14 days prior to T cell injection. Treatment with MAGE-A1 KLV/A2 TCR, MAGE-A1 SLF/A3 TCR, and MAGE-A1 VRF/C7 TCR-T cells demonstrated a major antitumor effect, with tumor cells becoming undetectable between day 3 and 8 after T cell infusion (Figures 6A–6D). Despite near-complete tumor eradication, tumor recurrence is observed after >25 days post TCR-T cell injection likely due to absence of the required human cytokine environment.

In conclusion, MAGE TCR-treated mice controlled tumor outgrowth up to 43 days after T cell infusion, confirming potent *in vivo* antitumor efficacy of our identified MAGE-specific TCRs.

DISCUSSION

In this study, we aimed to broaden the repertoire of TCRs targeting cancer testis antigens for potential clinical application. Using bioinformatics and immunopeptidomics, 31 *MAGE*-derived peptides were identified that were naturally presented in the most common HLA types. pHLA tetramer-positive $CD8^+$ T cells were isolated and the most promising T cell clones were selected based on various effectivity and safety screenings. Subsequently, to determine clinical potential of our identified T cell clones, TCRs were sequenced and transduced into peripheral blood-derived $CD8^+$ T cells. In total, we identified seven potent and unique MAGE-specific TCRs effectively targeting MAGE-A1, MAGE-A3, MAGE-A6, or MAGE-A9 expressing tumors in the context of HLA-A*01:01, -A*02:01, -A*03:01, -B*07:02, -B*35:01, or -C*07:02. Treatment with MAGE-A1-specific TCR-T cells resulted in total tumor eradication of MAGE-A1 expressing MM cells in an orthotopic xenograft mouse model, demonstrating strong antitumor reactivity of our identified TCRs *in vivo*. The identified set of MAGE-specific TCRs will enlarge the group of cancer patients that can be treated. The targeted HLA alleles, HLA-A*01:01, -A*02:01, -A*03:01, -B*07:02, -B*35:01, and -C*07:02, have relative allele frequencies in the worldwide population of 17%, 39%, 17%, 13%, 8%, and 21%, respectively. MAGE-A1, MAGE-A3, MAGE-A6, and MAGE-A9 are expressed in a variety of different tumor types, including in melanomas (MAGE-A1: 16%–20% in primary tumors and 48%–51% in metastases, MAGE-A3: 60%–75%), non-small cell lung carcinomas (MAGE-A1: 27%–46%, MAGE-A3: 38%–55%, MAGE-A6: 26%), breast carcinomas (MAGE-A9: 45%, MAGE-A3/A6: 10%–15%, MAGE-A1: 6%), ovarian carcinomas (MAGE-A1: 15%–53%, MAGE-A3: 36%–37%, MAGE-A9: 52%), colon carcinomas (MAGE-A1: 12%–30%, MAGE-A3: 20%–27%), and MM (MAGE-A1: <10%–26%, MAGE-A3/A6: 37%–41% in primary tumor and 77% in metastases).[18,19] Based on HLA typing and MAGE expression, approximately 33% of metastasized melanoma patients, 22%–35% of non-small cell lung cancer patients, 22%–48% of ovarian carcinoma patients, and 14%–26% of (relapsed) MM patients can be treated with the TCRs identified in this study.

An interesting observation was the strong anti-MM reactivity of the 6G4 (MAGE-A1 LTQ/A1) TCR-T cells, but limited reactivity to non-hematopoietic tumor cells. A potential explanation for this specific reactivity pattern could be lack of processing or presentation of LTQ peptide in HLA-A*01:01 by non-hematopoietic tumor cells, particularly since allo-HLA-A*01:01 reactive T cells efficiently recognized these cell lines, excluding reduced HLA-A*01:01 or adhesion molecule expression. In concordance with this, the LTQ peptide was eluted from MM cell line RPMI, confirming processing and presentation of this peptide in MM. However, the LTQ peptide was not eluted from non-hematopoietic-derived cells. The difference in processing and presentation of the LTQ peptide could be due to presence of immunoproteasomes in MM samples.[20–22] This immunoproteasome differs from the constitutive proteasome found in most other cell types, leading to enhanced ability to generate antigenic peptides. The immunoproteasome preferentially cleaves after hydrophobic residues. As the LTQ peptide (LTQDLVQEKYLEY) has a hydrophobic tyrosine at its C terminus, we hypothesize that the LTQ peptide is only presented on cells that contain immunoproteasomes like MMs.[23–25]

The TCR selection strategy from the T cell repertoire of HLA-mismatched individuals enables the selection of potent TCRs specific for self-antigens and can be used for variety of different targets.[26,27]

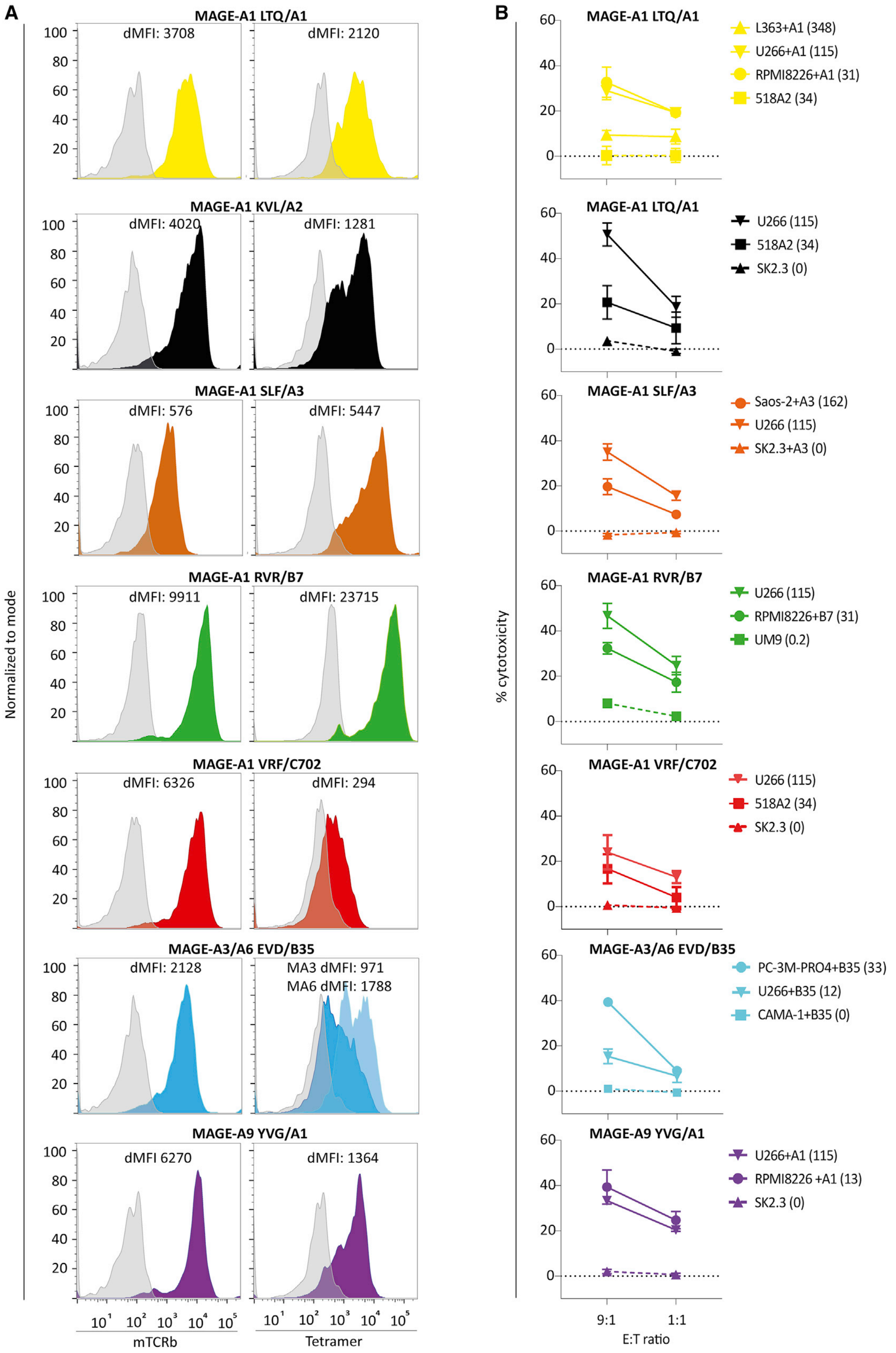

(legend on next page)

Our identified TCRs could potentially be included in an "off-the-shelf" library of TCRs. However, the use of allogeneic "off-the-shelf" TCRs for TCR gene therapy also comes with a toxicity risk. The importance of proper safety screenings during the selection process of MAGE-specific TCRs was underlined by two clinical trials, where MAGE-A3 targeting TCRs caused fatal toxicity. In the first study, two patients died of neurotoxicity caused by cross-reactivity with MAGE-A12 expressed in the brain.[15] To prevent unwanted recognition of any of the highly homologous MAGE-A family members, we screened our TCRs against a panel of Raji cells separately Td with all MAGE-A family members. The TCRs recognizing MAGE-A family members with an unsafe expression profile were excluded from further analysis. In a second clinical trial, unexpected reactivity against a homologous peptide derived from titin resulted in fatal cardiac toxicity of two patients.[14] This titin peptide was as much as four amino acids different from the MAGE-A3 peptide.[28] To limit the risk to induce off-target toxicity, we included an MAGE-negative cell line and healthy cell subset panel consisting of cells from different origins, and only those T cell clones identified as safe were kept in the selection. However, as not all genes will be expressed by the included cell lines and healthy cell subsets, potential cross-reactivities could have remained unidentified. In future preclinical studies, identified TCRs should additionally be screened against a combinatorial peptide library to even further reduce the risk of cross-reactivity against unrelated peptides.[16,29] In addition, to reduce potential toxicity risks of TCRs in clinical trials, a suicide switch could be considered, as suicide switches would enable immediate elimination of the transfused T cells upon signs of unexpected toxicity.[30]

In summary, we used a high-throughput method to identify a set of MAGE-specific TCRs valuable for TCR gene therapeutic strategies. This resulted in the identification of MAGE-A1-, MAGE-A3-, MAGE-A6-, and MAGE-A9-specific TCRs targeting peptides in the context of HLA-A*01:01, -A*02:01, -A*03:01, -B*07:02, -B*35:01, or -C*07:02. The identified TCRs can be included in an "off-the-shelf" TCR library that enables treatment of cancer patients with prior generated TCR constructs. Multiple TCRs could then be selected for a personalized and multi-antigen-targeting T-cell therapy.

MATERIAL AND METHODS

Cell culture

Primary CD8⁺ T cells were isolated from PBMCs by magnetic-activated cell sorting (MACS) using anti-CD8 MicroBeads (Miltenyi Biotec). CD8⁺ T cells (0.3×10^6 cells/mL) used for TCR transduction were stimulated with irradiated autologous PBMCs (1×10^6 cells/well), and 0.8 μg/mL PHA and cultured in T cell medium (TCM) containing Iscove modified Dulbecco medium (IMDM) (Lonza) supplemented with 5% fetal bovine serum (FBS) (Thermo Fisher Scientific), 5% human serum (Sanquin), 100 IU/mL interleukin (IL)-2 (Novartis Pharma), 1.5% L-glutamine (Lonza), and 1% penicillin/streptomycin (pen/strep) (Lonza). T cells for the healthy subset panel were generated by stimulating PBMCs with 1% TransAct (Miltenyi Biotec) and cultured in TCM. Tumor cell lines and Epstein-Barr Virus transformed B lymphoblastoid cell lines (EBV-LCLs) were cultured in IMDM supplemented with 10% FBS, 1.5% L-glutamine, and 1% pen/strep. Fibroblasts and keratinocytes were obtained according to standard procedures. Fibroblasts were cultured in DMEM supplemented with 10% FBS, 1.5% L-glutamine, and 1% pen/strep. Keratinocytes were cultured in keratinocyte serum-free medium (Gibco) supplemented with 0.2 ng/mL epidermal growth factor (Promega), 30 μg/mL bovine pituitary extract (Thermo Fisher Scientific), 1.5% L-glutamine, and 1% pen/strep. To obtain immature dendritic cells (imDC) and mature dendritic cells (mDC), CD14⁺ cells were isolated from PBMCs by MACS using anti-CD14 CliniMACS beads (Miltenyi Biotec). CD14⁺ cells were stimulated with granulocyte-macrophase colony-stimulating factor (GM-CSF) (100 ng/mL) and IL-4 (500 IU/mL) for 2 days to generate imDC and subsequently with GM-CSF (100 ng/mL), tumor necrosis factor-α (10 ng/mL), IL-1b (10 ng/mL), IL-6 (10 ng/mL), PGE-2 (1 μg/mL), and IFN-γ (500 IU/mL) for 3 days to generate mDC. Early-passage melanoma cell lines were obtained from the Department of Medical Oncology, Leiden University Medical Center, and cultured in DMEM (Thermo Fisher Scientific) containing nonessential amino acids supplemented with 7.5% FBS, 1.5% L-glutamine, and 1% pen/strep. The Leiden University Medical Center ethical review board approved use of all the human material in this study (approval number B16.039). Materials were obtained after written informed consent in accordance with the Declaration of Helsinki.

Peptide identification and pHLA tetramer generation

Peptides were identified from resected ovarian carcinomas (OvaL1, OvaL10, and OvaL11), MM cell lines (U266, UM9, and RPMI8226) and a prostate cell line (C4-2B4) that expressed at least one of the selected MAGE family members. The resected ovarian carcinomas consisted of residual surgical material and were collected anonymously. The carcinoma samples were sliced into small pieces and

Figure 4. TCR expression and cytotoxicity of transduced primary CD8⁺ T cells against malignant cell lines

(A) The TCR expression of selected TCRs (6G4 (MAGE-A1 LTQ/A1), 4F7 (MAGE-A1 KVL/A2), 3H4 (MAGE-A1 SLF/A3), 3G2 (MAGE-A1 RVR/B7), 10C1 (MAGE-A1 VRF/C7), 2H9 (MAGE-A3/A6 EVD/B35), and 2D8 (MAGE-A9 YVG/A1) was determined after transduction in primary CD8⁺ T cells. TCR-T cells were stained with mTCRβ APC (left) and pHLA tetramer PE (right). In the graph, the delta mean fluorescent intensity (MFI) (sample MFI – control MFI) of the tetramer and mTCRβ stain are depicted. Untransduced or CMV TCR-T cells were included as negative control (depicted in gray). (B) Cytotoxicity of the TCR-T cells against multiple tumor cell lines was determined by 6-h ^{51}Cr-release assay with E:T ratio of 9:1 or 1:1. T cell clones were stimulated with tumor cell lines of different origin, including multiple myeloma (L363, U266, RPMI8226, UM9), melanoma (518A2, SK2.3), osteosarcoma cell line Saos-2, mammary carcinoma cell line CAMA-1, and prostate carcinoma cell line PC-3M-PRO4. Target cells naturally express target HLA or were transduced with target HLA alleles (+HLA). The *MAGE*-gene expression levels, measured by qPCR, are depicted between brackets as percentage relative to HKGs. CMV TCR-T cells were included as negative control and allo-HLA reactive T cell clones as positive control for HLA expression and killing capacity. Values and error bars represent means and standard deviations of technical triplicates, and experiments are representative of at least two independent experiments.

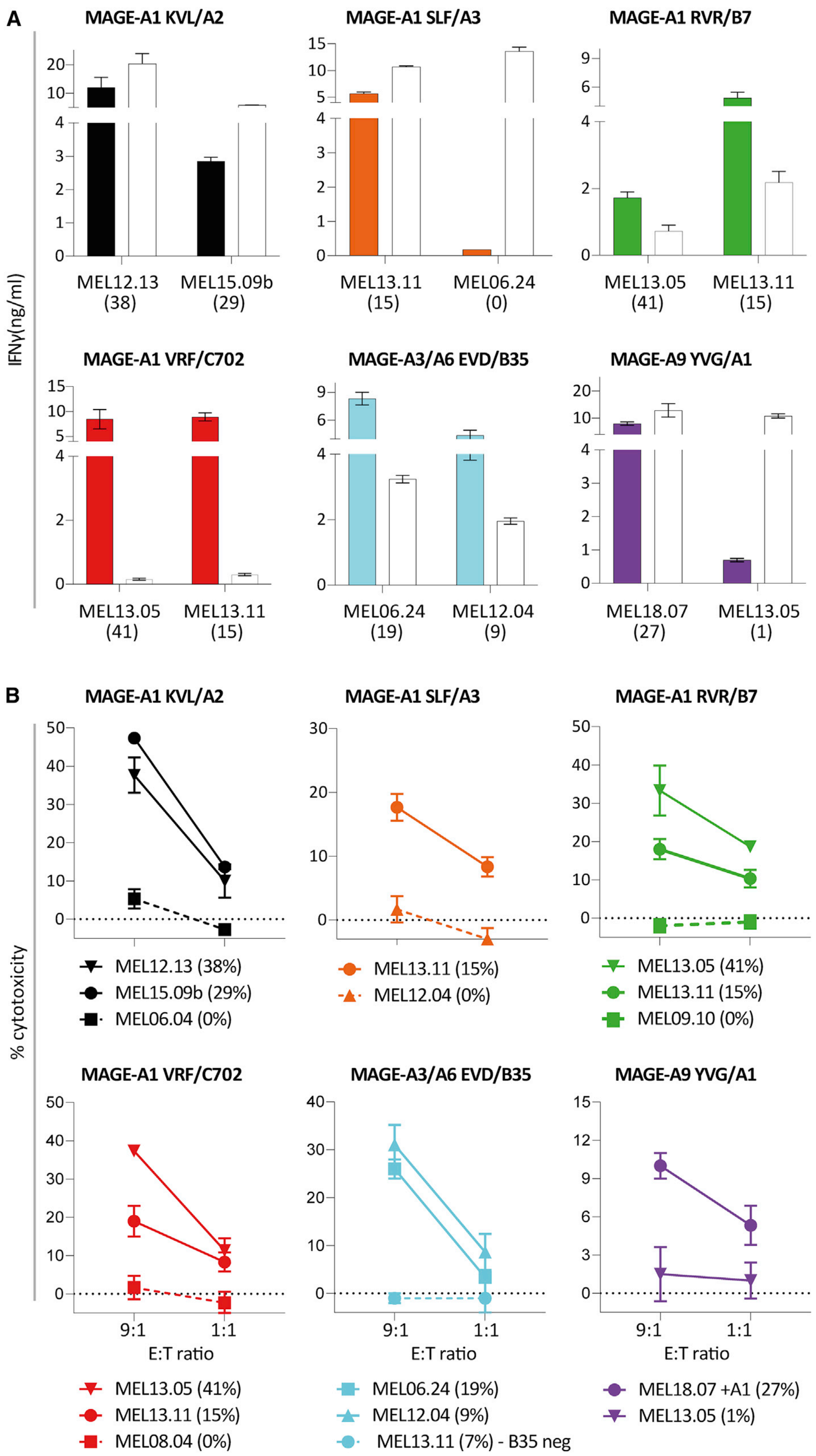

(legend on next page)

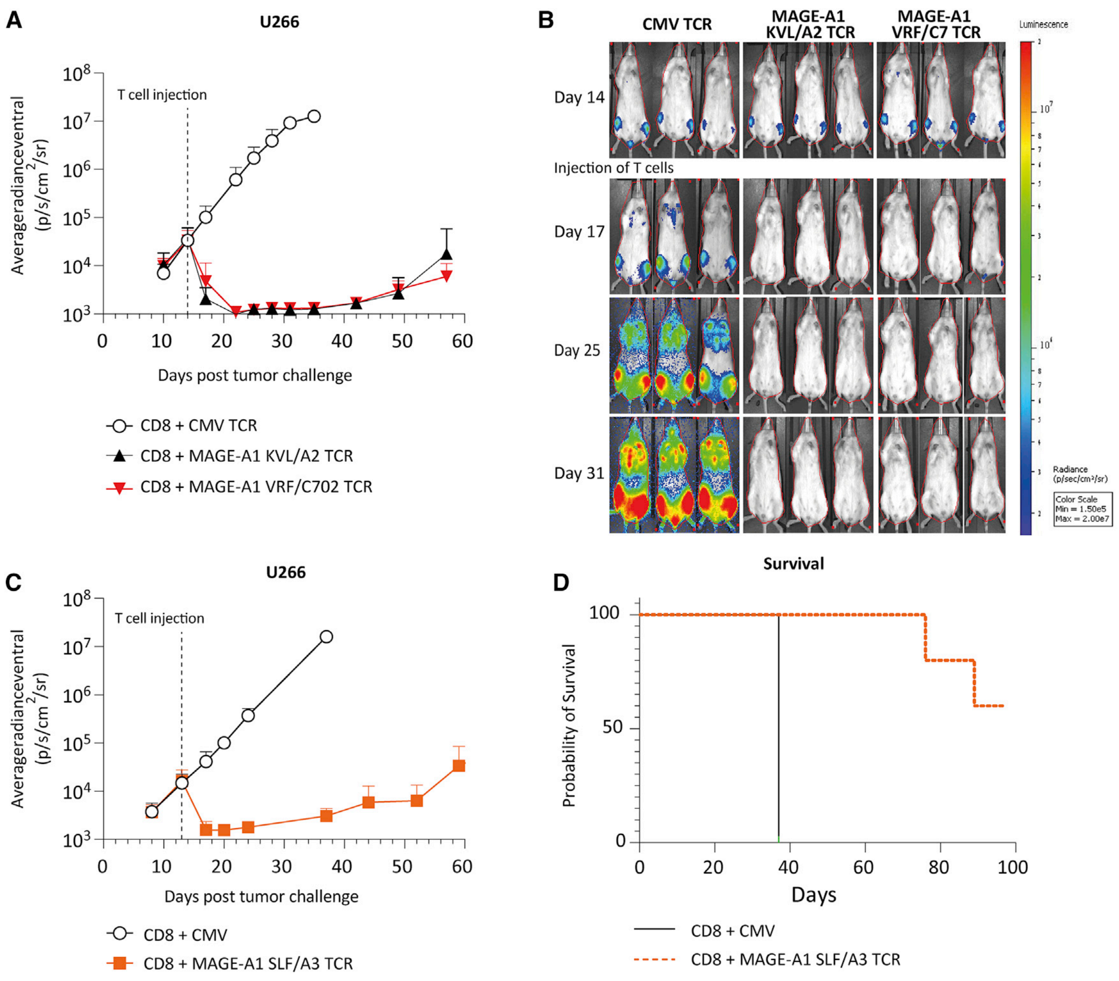

Figure 6. *In vivo* antitumor efficacy of MAGE-A1 specific HLA-A2, -A3, and -C7 restricted TCRs

NSG mice engrafted with 2 × 10^6 U266 cells were i.v. injected with 5 × 10^6 TCR-T cells 14 days after tumor injection. T cells were transduced with the 4F7 (MAGE-A1 KVL/A2) TCR, 3H4 (MAGE-A1 SLF/A3) TCR, 10C1 (MAGE-A1 VRF/C7) TCR, or CMV (pp65 NLV/A2) TCR and enriched for mTCRβ expression by MACS. Tumor growth was visualized by bioluminescence imaging 1–2 times per week. (A) Mean of average tumor outgrowth of the dorsal and ventral side of 6 × 4F7 TCR-T cell (black), 6 × 10C1 TCR-T cell (red), and 4 × CMV TCR-T cell (white) treated mice. (B) Tumor outgrowth of representative individual 4F7 TCR-T cell, 10C1 TCR-T cell, and CMV TCR-T cell treated mice measured on the ventral side. (C) Mean of average tumor outgrowth of the dorsal and ventral side of 5 × 3H4 TCR-T cell (red) and 4 × CMV TCR-T cell (white). (D) Kaplan-Meier plot of 3H4 TCR-T cell and CMV TCR-T cell treated mice.

dead, clotted, or non-tumor material was removed during visual inspection. The sliced tumor pieces were added to a C-tube (Miltenyi Biotec) with ice cold buffer and Complete Protease Inhibitor (Sigma-Aldrich), but without detergent, to prevent unwanted protease activity. The tumor pieces were dissociated until an almost homogeneous cell solution by using a gentleMACS (Miltenyi Biotec) procedure and benzonase (Merck) (125 IU/mL) was added to remove DNA/RNA complexes during lysis. To identify peptides from the resected ovarian carcinomas, MM cell lines, and prostate cell line, peptide elution experiments were performed followed by high-performance liquid chromatography (HPLC) fractionation and mass spectrometry (MS) analysis as previously described.[31] In short, cells of

Figure 5. Reactivity of the MAGE-specific TCRs against early-passage melanoma cell lines

(A) To determine recognition of early-passage melanoma cell lines (passage ≤10), TCR-T cells were stimulated. All melanomas (MEL) naturally expressed the HLA of interest except for MEL18.07 that was transduced with HLA-A*01:01. IFN-γ production, as measured by ELISA, was determined after an overnight stimulation in at least two independent experiments. An allo-HLA reactive T cell clone was included for each target HLA as a positive control for HLA expression and stimulatory capacity of target cells. The *MAGE*-gene expression levels, measured by qPCR, are depicted between brackets as percentage relative to HKGs. Values and error bars represent means and standard deviations of technical duplicates. (B) Cytotoxicity of the TCR-T cells was analyzed by a 6-h ^{51}Cr-release assay in at least two independent experiments. In Figure S5, the positive (allo-HLA reactive clones) and negative (CMV TCR) are shown. Values and error bars represent means and standard deviations of technical triplicates.

interest were lysed and peptide-HLA complexes were purified by immunoaffinity using the anti-HLA-I W6/32 antibody. Peptides were separated from the HLA allele and peptide-containing fractions were obtained by size filtration. Subsequently, fractions were separated by strong cation exchange chromatography and freeze dried. Peptide fractions were lyophilized, dissolved in 95/3/0.1 water/acetonitrile/formic acid v/v/v, and subsequently analyzed with nanoHPLC-MS/MS. Peptide and protein identification from tandem mass spectra was performed by proteome discoverer version 2.1 (Thermo Fischer Scientific) using the mascot node and the UniProt Homo Sapiens database. Synthetic peptides were ordered for the potential target peptides when meeting the following criteria: (1) peptides are derived from the selected candidate genes; (2) bind in HLA-A*01:01, -A*02:01, -A*03:01, -A*11:01, -A*24:02, -B*07:02, -B*35:01, -C*07:01, or -C*07:02 according to NETMHC version 4.0; (3) have minimal Mascot Ion score ≥ 30; (4) are ranked 1 peptides; and (5) peptide sequence is unique for the candidate gene. Synthetic peptides were generated in-house using standard Fmoc chemistry. Peptide sequences were validated by matching the tandem mass spectra of the eluted peptides with the tandem mass spectra of the synthetic peptides. To generate monomers recombinant HLA-A*01:01, -A*02:01, -A*03:01, -A*08:01, -A*24:02, -B*07:02, -B*35:01, -C*07:01, and -C*07:02 heavy chains (HCs) and human beta-2 microglobulin (B2M) were produced in-house in *Escherichia coli*. PE-labeled pHLA tetramers were generated from the monomers as previously described.[32]

Isolation of MAGE-specific T cell clones

After informed consent was collected, PBMCs from healthy individuals were isolated from complete buffy coats (Sanquin) using Ficoll gradient centrifugation. pHLA tetramer-positive T cells were isolated from 250–750 $\times$ 10^6 PBMCs from donors negative for the HLA of interest, as previously described.[12] In brief, PBMCs were stained with PE-labeled pHLA tetramers for 10 min at 37°C or 1 h at 4°C. Subsequently, MACS was performed using anti-PE Microbeads (Miltenyi Biotec). The positive fraction was stained with Alexa Fluor700-conjugated anti-CD8 (catalog MHCD0829) (Invitrogen), FITC-conjugated anti-CD4 (catalog 555346) (BD Pharmingen), anti-CD14 (catalog 555397) (BD Pharmingen), and anti-CD19 (catalog 555412) (BD Biosciences). pHLA tetramer-positive $CD8^+$ T cells were single-cell sorted using an Aria III cell sorter (BD Biosciences) in 96-well round-bottom culture plates (Greiner Bio-one) containing irradiated PBMCs (50,000 cells/well) (3500RAD) and EBV-LCLs (5000 cells/well) (5000RAD) in 100 μL TCM supplemented with 0.8 μg/mL phytohemagglutinin (PHA) (Thermo Fisher Scientific). Expanding T cell clones were used in functional assays 7–14 days after stimulation. pHLA tetramer staining was performed using PE-conjugated pHLA tetramers for 15 min at 37°C and fluorescence was measured on an LSRII (BD Biosciences) and analyzed using Diva (BD Biosciences) or FlowJo software (TreeStar).

T cell recognition assay

Target cell recognition was determined by co-culture of 5,000 T cells in effector:target (E:T) ratio of 1:2 or 1:4 in 60 μL TCM per well in 384-well flat-bottom culture plates (Greiner Bio-one). To induce HLA expression prior to functional analysis, early-passage melanoma cell lines, fibroblasts, and keratinocytes were treated with 100 IU/mL IFN-γ for 48 h. In peptide titration assays, target cells were pre-incubated for at least 30 min at 37°C with titrated peptide concentrations starting at 1 μM. After overnight co-culture, target cell recognition was determined by measuring IFN-γ production in the supernatant by ELISA (Sanquin/Invitrogen/Diaclone). Supernatants were diluted 5, 25, or 125 times to calculate concentrations based on the linear part of the standard curve. The Hamilton Microlab STAR Liquid Handling System (Hamilton company) was used to transfer supernatants from the culture plates to the high-binding 384-well ELISA plates (Greiner Bio-one). ELISA plates were washed using the Zoom HT LB 920 Microplate Washer (Berthold).

T cell-mediated cytotoxicity was measured using ^{51}Cr-release experiments. Target cells were labeled for 1 h at 37°C with 50 μCi $Na_2{}^{51}CrO_4$ (PerkinElmer). Subsequently, target cells were washed and co-cultured with T cells at various E:T ratios for 6 h at 37°C in 96-well round-bottom culture plates (Greiner Bio-one). After incubation, supernatant was harvested, transferred to Lumaplates (Microbeta 2, PerkinElmer), and dried overnight. Spontaneous and maximum ^{51}Cr-release was determined using TCM alone and TCM containing 1% Triton-X 100 (Sigma-Aldrich), respectively. ^{51}Cr release was measured in counts per minute (CPM) using a 2450 Microbeta2 plate counter (PerkinElmer). Target cell killing was calculated by the following formula: (average CPM of the sample − average CPM spontaneous release)/(average CPM maximal release − average CPM spontaneous release)*100.

TCR constructs and retroviral transduction

TCR usage was determined as previously described with minor modifications.[33] In short, T cells were lysed (ReliaPromega kit), mRNA was isolated, and TCR cDNA was generated using reverse primers in the TCR constant alfa (TCR-α) and beta (TCR-β) regions, SMARTScribe Reverse Transcriptase (Takara, Clontech), and an SA.rt template switching oligo forward primer.[34] Barcoded TCR PCR product was generated in two rounds of PCR. In the first PCR, TCR-α and TCR-β products were generated, in a second PCR the first PCR product was used to include a barcode sequence that allowed discrimination between TCRs of different T cell clones. PCR products of different T cell clones were pooled, after which TCR sequences were identified by HiSeq (GenomeScan). The TCR sequences were analyzed using MiXCR software package and the ImMunoGeneTics (IMGT) database.[35] The variable TCR-α and TCR-β fragments of the different MAGE-specific TCRs and CMV TCR specific for the NVLPMVATV peptide binding in HLA-A*02:01 were codon optimized and combined with codon-optimized and cysteine-modified murine TCR-α and -β constant domains, TCR chains were linked by P2A sequence and cloned into MP71 retroviral vector.[26]

TCR gene transfer

Transfection of the TCR constructs was performed with plasmid DNA, Fugene HD transfection reagent (Promega), and optimum I

medium (Invitrogen Gibco) in Phoenix-A cells. Phoenix-A cells (ATCC) were transfected, virus supernatant was harvested 48 h after transfection, and stored at −80°C. On day 2 after T cell stimulation, retroviral supernatants were added to 24-well non-tissue-culture-treated plates (Greiner Bio-One) precoated with retronectin (Takara) and blocked with 2% human serum albumin (Sanquin). Retroviral supernatant was spun down for 20 min, 3,000 RPM at 4°C, after which the virus supernatant was removed and 0.3 × 106 CD8⁺ T cells were added for overnight culture. After overnight culture, T cells were transferred to 24-well culture plates (Costar) and 5 days after transduction T cells were enriched by MACS using APC-conjugated anti-mouse TCR-β constant domain (mTCRβ) (BD Biosciences) and anti-APC Microbeads (Miltenyi Biotec). pHLA tetramer staining was performed to confirm TCR-cell surface expression. TCR-T cells were used in functional screenings 9–12 days after isolation.

Quantitative RT-PCR

Total RNA was isolated from 0.1–5 × 10^6 target cells using the Reliaprep RNA cell mini prep system according to manufacturer's protocol (Promega). Total RNA was converted to cDNA using Moloney murine leukemia virus reverse transcriptase and oligo (dT) primer (Invitrogen by Thermo Fisher Scientific). qPCR was performed using Fast Start TaqDNA Polymerase (Roche) and EvaGreen (Biotium). Gene expression was measured on the Lightcycler 480 (Roche) using forward and reverse primers listed in Table S3. Target gene expression was calculated relative to the average expression of housekeeping genes (HKGs): GUSB, PSMB4, and VPS29.

In vivo antitumor reactivity of MAGE-A1-specific TCRs

Female NSG mice (NOD.Cg-Prkdc(scid)Il2rg(tm1Wjl)/SzJ, The Jackson Laboratory) were injected intravenously (i.v.) with 2 × 10^6 U266 MM cells. U266 cells were previously transduced to express Luciferase-TdTomato Red and enriched for >98% purity. Tumor growth was measured 1 to 2 times per week after subcutaneous injection of 150 μL 7.5 mM D-luciferine (Cayman Chemical Co.) using a charge-coupled device camera (IVIS spectrum, PerkinElmer). On day 14 post engraftment, mice were injected i.v. with 5 × 10^6 T cells that were transduced with 4F7-TCR (MAGE-A1 KVL/A2) (n = 6), 3H4-TCR (MAGE-A1 SLF/A3) (n = 5), 10C1-TCR (MAGE-A1 VRF/C7) (n = 6), or irrelevant CMV TCR (pp65 NLV/A2) (n = 4). TCR-T cells were enriched for mTCRβ expression by MACS before infusion and injected 7 days after an additional restimulation. This *in vivo* study was performed in accordance with Dutch law for animal experiments and approved by the national Ethical Committee for Animal Research (AVD116002017891).

DATA AVAILABILITY STATEMENT

All raw data are available upon request.

SUPPLEMENTAL INFORMATION

Supplemental information can be found online at https://doi.org/10.1016/j.omto.2022.11.007.

ACKNOWLEDGMENTS

The authors gratefully acknowledge the Flow cytometry Core Facility (FCF) of Leiden University Medical Center (LUMC) in Leiden, the Netherlands (https://www.lumc.nl/research/facilities/fcf), coordinated by Dr. K. Schepers and M. Hameetman, run by the FCF Operators E.F.E de Haas, J.P. Jansen, D.M. Lowie, S. van de Pas, and G.I.J. Reyneveld (Directors: Prof. F.J.T. Staal and Prof. J.J.M. van Dongen) for cell sorting assistance. This study was funded by Bellicum Pharmaceuticals (unrestricted grant) and Health-Holland (grant number LSHM17002).

AUTHOR CONTRIBUTIONS

M.A.J.d.R. conceived the research, designed the experiments, performed experiments, analyzed data, and performed manuscript writing and editing. D.M.v.d.S., D.F.G.R., A.W., R.S.H., A.H.d.R, M.H.M., T.L.A.W., and M.G.D.K. performed experiments and data analysis. E.M.E.V. provided samples and scientific guidance. P.A.v.V. performed experiments and provided scientific guidance. J.H.F.F. and M.H.M.H. provided scientific guidance by experimental design, data analysis, and manuscript editing. M.H.M.H. provided supervision and funding acquisition. All the authors reviewed the manuscript.

DECLARATION OF INTERESTS

The authors report no competing interests.

REFERENCES

1. GTEx Consortium (2013). The genotype-tissue expression (GTEx) project. Nat. Genet. *45*, 580–585. https://doi.org/10.1038/ng.2653.
2. Fawcett, D.W., Leak, L.V., and Heidger, P.M., Jr. (1970). Electron microscopic observations on the structural components of the blood-testis barrier. J. Reprod. Fertil. Suppl. *10*, 105–122.
3. Schaller, J., Glander, H.J., Ladusch, M., Westhoff, U., and Grosse-Wilde, H. (1993). Lack of HLA-molecules on human spermatozoa and in seminal plasma. Andrologia *25*, 77–81. https://doi.org/10.1111/j.1439-0272.1993.tb02686.x.
4. Kowalik, I., Kurpisz, M., Jakubowiak, A., Janecki, A., Lukaszyk, A., and Szymczynski, G. (1989). Evaluation of HLA expression on gametogenic cells isolated from human testis. Andrologia *21*, 237–243.
5. Gotter, J., Brors, B., Hergenhahn, M., and Kyewski, B. (2004). Medullary epithelial cells of the human thymus express a highly diverse selection of tissue-specific genes colocalized in chromosomal clusters. J. Exp. Med. *199*, 155–166. https://doi.org/10.1084/jem.20031677.
6. Theobald, M., Biggs, J., Hernández, J., Lustgarten, J., Labadie, C., and Sherman, L.A. (1997). Tolerance to p53 by A2.1-restricted cytotoxic T lymphocytes. J. Exp. Med. *185*, 833–841. https://doi.org/10.1084/jem.185.5.833.
7. Grob, J.J., Mortier, L., D'Hondt, L., Grange, F., Baurain, J., Dréno, B., Lebbe, C., Robert, C., Dompmartin, A., Neyns, B., et al. (2017). Safety and immunogenicity of MAGE-A3 cancer immunotherapeutic with dacarbazine in patients with MAGE-A3-positive metastatic cutaneous melanoma: an open phase I/II study with a first assessment of a predictive gene signature. ESMO Open *2*, e000203. https://doi.org/10.1136/esmoopen-2017-000203.
8. Vansteenkiste, J.F., Cho, B.C., Vanakesa, T., De Pas, T., Zielinski, M., Kim, M.S., Jassem, J., Yoshimura, M., Dahabreh, J., Nakayama, H., et al. (2016). Efficacy of the MAGE-A3 cancer immunotherapeutic as adjuvant therapy in patients with resected MAGE-A3-positive non-small-cell lung cancer (MAGRIT): a randomised, double-blind, placebo-controlled, phase 3 trial. Lancet Oncol. *17*, 822–835. https://doi.org/10.1016/S1470-2045(16)00099-1.

9. Sadovnikova, E., and Stauss, H.J. (1996). Peptide-specific cytotoxic T lymphocytes restricted by nonself major histocompatibility complex class I molecules: reagents for tumor immunotherapy. Proc. Natl. Acad. Sci. USA *93*, 13114–13118. https://doi.org/10.1073/pnas.93.23.13114.

10. Amir, A.L., van der Steen, D.M., van Loenen, M.M., Hagedoorn, R.S., de Boer, R., Kester, M.D.G., de Ru, A.H., Lugthart, G.J., van Kooten, C., Hiemstra, P.S., et al. (2011). PRAME-specific Allo-HLA-restricted T cells with potent antitumor reactivity useful for therapeutic T-cell receptor gene transfer. Clin. Cancer Res. *17*, 5615–5625. https://doi.org/10.1158/1078-0432.CCR-11-1066.

11. Gezgin, G., Luk, S.J., Cao, J., Dogrusöz, M., van der Steen, D.M., Hagedoorn, R.S., Krijgsman, D., van der Velden, P.A., Field, M.G., Luyten, G.P.M., et al. (2017). PRAME as a potential target for immunotherapy in metastatic uveal melanoma. JAMA Ophthalmol. *135*, 541–549. https://doi.org/10.1001/jamaophthalmol.2017.0729.

12. Jahn, L., Hombrink, P., Hagedoorn, R.S., Kester, M.G.D., van der Steen, D.M., Rodriguez, T., Pentcheva-Hoang, T., de Ru, A.H., Schoonakker, M.P., Meeuwsen, M.H., et al. (2017). TCR-based therapy for multiple myeloma and other B-cell malignancies targeting intracellular transcription factor BOB1. Blood *129*, 1284–1295. https://doi.org/10.1182/blood-2016-09-737536.

13. Luk, S.J., van der Steen, D.M., Hagedoorn, R.S., Jordanova, E.S., Schilham, M.W., Bovée, J.V., Cleven, A.H., Falkenburg, J.F., Szuhai, K., and Heemskerk, M.H. (2018). PRAME and HLA Class I expression patterns make synovial sarcoma a suitable target for PRAME specific T-cell receptor gene therapy. Oncoimmunology *7*, e1507600. https://doi.org/10.1080/2162402X.2018.1507600.

14. Linette, G.P., Stadtmauer, E.A., Maus, M.V., Rapoport, A.P., Levine, B.L., Emery, L., Litzky, L., Bagg, A., Carreno, B.M., Cimino, P.J., et al. (2013). Cardiovascular toxicity and titin cross-reactivity of affinity-enhanced T cells in myeloma and melanoma. Blood *122*, 863–871. https://doi.org/10.1182/blood-2013-03-490565.

15. Morgan, R.A., Chinnasamy, N., Abate-Daga, D., Gros, A., Robbins, P.F., Zheng, Z., Dudley, M.E., Feldman, S.A., Yang, J.C., Sherry, R.M., et al. (2013). Cancer regression and neurological toxicity following anti-MAGE-A3 TCR gene therapy. J. Immunother. *36*, 133–151. https://doi.org/10.1097/CJI.0b013e3182829903.

16. Bijen, H.M., van der Steen, D.M., Hagedoorn, R.S., Wouters, A.K., Wooldridge, L., Falkenburg, J.H.F., and Heemskerk, M.H.M. (2018). Preclinical strategies to identify off-target toxicity of high-affinity TCRs. Mol. Ther. *26*, 1206–1214. https://doi.org/10.1016/j.ymthe.2018.02.017.

17. Doyle, J.M., Gao, J., Wang, J., Yang, M., and Potts, P.R. (2010). MAGE-RING protein complexes comprise a family of E3 ubiquitin ligases. Mol. Cell *39*, 963–974. https://doi.org/10.1016/j.molcel.2010.08.029.

18. Weon, J.L., and Potts, P.R. (2015). The MAGE protein family and cancer. Curr. Opin. Cell Biol. *37*, 1–8. https://doi.org/10.1016/j.ceb.2015.08.002.

19. Kruit, W.H.J., Suciu, S., Dreno, B., Mortier, L., Robert, C., Chiarion-Sileni, V., Maio, M., Testori, A., Dorval, T., Grob, J.J., et al. (2013). Selection of immunostimulant AS15 for active immunization with MAGE-A3 protein: results of a randomized phase II study of the European organisation for research and treatment of cancer melanoma group in metastatic melanoma. J. Clin. Oncol. *31*, 2413–2420. https://doi.org/10.1200/JCO.2012.43.7111.

20. Zerfas, B.L., Maresh, M.E., and Trader, D.J. (2020). The immunoproteasome: an emerging target in cancer and autoimmune and neurological disorders. J. Med. Chem. *63*, 1841–1858. https://doi.org/10.1021/acs.jmedchem.9b01226.

21. Rock, K.L., and Goldberg, A.L. (1999). Degradation of cell proteins and the generation of MHC class I-presented peptides. Annu. Rev. Immunol. *17*, 739–779. https://doi.org/10.1146/annurev.immunol.17.1.739.

22. Rock, K.L., Gramm, C., Rothstein, L., Clark, K., Stein, R., Dick, L., Hwang, D., and Goldberg, A.L. (1994). Inhibitors of the proteasome block the degradation of most cell proteins and the generation of peptides presented on MHC class I molecules. Cell *78*, 761–771. https://doi.org/10.1016/s0092-8674(94)90462-6.

23. Aki, M., Shimbara, N., Takashina, M., Akiyama, K., Kagawa, S., Tamura, T., Tanahashi, N., Yoshimura, T., Tanaka, K., and Ichihara, A. (1994). Interferon-gamma induces different subunit organizations and functional diversity of proteasomes. J. Biochem. *115*, 257–269. https://doi.org/10.1093/oxfordjournals.jbchem.a124327.

24. Driscoll, J., Brown, M.G., Finley, D., and Monaco, J.J. (1993). MHC-linked LMP gene products specifically alter peptidase activities of the proteasome. Nature *365*, 262–264. https://doi.org/10.1038/365262a0.

25. Gaczynska, M., Rock, K.L., and Goldberg, A.L. (1993). Gamma-interferon and expression of MHC genes regulate peptide hydrolysis by proteasomes. Nature *365*, 264–267. https://doi.org/10.1038/365264a0.

26. Meeuwsen, M.H., Wouters, A.K., Jahn, L., Hagedoorn, R.S., Kester, M.G.D., Remst, D.F.G., Morton, L.T., van der Steen, D.M., Kweekel, C., de Ru, A.H., et al. (2022). A broad and systematic approach to identify B cell malignancy-targeting TCRs for multi-antigen-based T cell therapy. Mol. Ther. *30*, 564–578. https://doi.org/10.1016/j.ymthe.2021.08.010.

27. van Amerongen, R.A., Hagedoorn, R.S., Remst, D.F.G., Assendelft, D.C., van der Steen, D.M., Wouters, A.K., van de Meent, M., Kester, M.G.D., de Ru, A.H., Griffioen, M., et al. (2022). WT1-specific TCRs directed against newly identified peptides install antitumor reactivity against acute myeloid leukemia and ovarian carcinoma. J. Immunother. Cancer *10*, e004409. https://doi.org/10.1136/jitc-2021-004409.

28. Cameron, B.J., Gerry, A.B., Dukes, J., Harper, J.V., Kannan, V., Bianchi, F.C., Grand, F., Brewer, J.E., Gupta, M., Plesa, G., et al. (2013). Identification of a Titin-derived HLA-A1-presented peptide as a cross-reactive target for engineered MAGE A3-directed T cells. Sci. Transl. Med. *5*, 197ra103. https://doi.org/10.1126/scitranslmed.3006034.

29. Szomolay, B., Liu, J., Brown, P.E., Miles, J.J., Clement, M., Llewellyn-Lacey, S., Dolton, G., Ekeruche-Makinde, J., Lissina, A., Schauenburg, A.J., et al. (2016). Identification of human viral protein-derived ligands recognized by individual MHCI-restricted T-cell receptors. Immunol. Cell Biol. *94*, 573–582. https://doi.org/10.1038/icb.2016.12.

30. Di Stasi, A., Tey, S.K., Dotti, G., Fujita, Y., Kennedy-Nasser, A., Martinez, C., Straathof, K., Liu, E., Durett, A.G., Grilley, B., et al. (2011). Inducible apoptosis as a safety switch for adoptive cell therapy. N. Engl. J. Med. *365*, 1673–1683. https://doi.org/10.1056/NEJMoa1106152.

31. van der Lee, D.I., Reijmers, R.M., Honders, M.W., Hagedoorn, R.S., de Jong, R.C., Kester, M.G., van der Steen, D.M., de Ru, A.H., Kweekel, C., Bijen, H.M., et al. (2019). Mutated nucleophosmin 1 as immunotherapy target in acute myeloid leukemia. J. Clin. Invest. *129*, 774–785. https://doi.org/10.1172/JCI97482.

32. Burrows, S.R., Kienzle, N., Winterhalter, A., Bharadwaj, M., Altman, J.D., and Brooks, A. (2000). Peptide-MHC class I tetrameric complexes display exquisite ligand specificity. J. Immunol. *165*, 6229–6234. https://doi.org/10.4049/jimmunol.165.11.6229.

33. van Bergen, C.A.M., van Luxemburg-Heijs, S.A.P., de Wreede, L.C., Eefting, M., von dem Borne, P.A., van Balen, P., Heemskerk, M.H.M., Mulder, A., Claas, F.H.J., Navarrete, M.A., et al. (2017). Selective graft-versus-leukemia depends on magnitude and diversity of the alloreactive T cell response. J. Clin. Invest. *127*, 517–529. https://doi.org/10.1172/JCI86175.

34. Koning, M.T., Kiełbasa, S.M., Boersma, V., Buermans, H.P.J., van der Zeeuw, S.A.J., van Bergen, C.A.M., Cleven, A.H.G., Kluin, P.M., Griffioen, M., Navarrete, M.A., and Veelken, H. (2017). Artisan PCR: rapid identification of full-length immunoglobulin rearrangements without primer binding bias. Br. J. Haematol. *178*, 983–986. https://doi.org/10.1111/bjh.14180.

35. Lefranc, M.P. (2003). IMGT, the international ImMunoGeneTics database. Nucleic Acids Res. *31*, 307–310. https://doi.org/10.1093/nar/gkg085.

OMTO, Volume *28*

Supplemental information

A library of cancer testis specific T cell receptors for T cell receptor gene therapy

Marije A.J. de Rooij, Dennis F.G. Remst, Dirk M. van der Steen, Anne K. Wouters, Renate S. Hagedoorn, Michel G.D. Kester, Miranda H. Meeuwsen, Tassilo L.A. Wachsmann, Arnoud H. de Ru, Peter A. van Veelen, Els M.E. Verdegaal, J.H. Frederik Falkenburg, and Mirjam H.M. Heemskerk

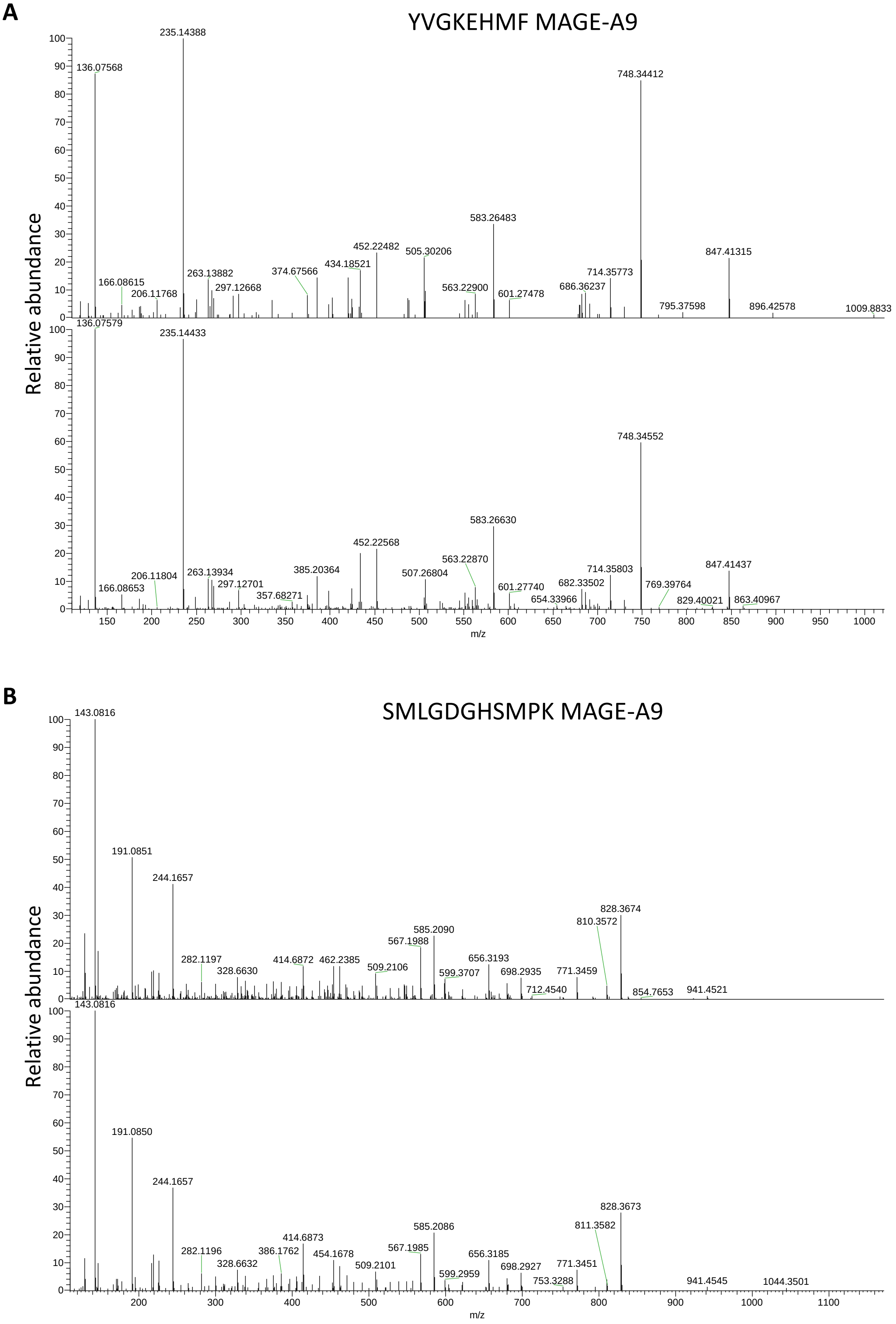

Figure S1. Peptide sequence validation of matching tandem mass spectra of eluted (top) and synthetically (bottom) generated peptides. Two examples of matching tandem mass spectra of (A) the YVGKEHMF peptide presented in HLA-A*24:02 and (B) the SMLGDGHSMPK peptide presented in HLA-A*03:01. Both depicted peptides are derived from MAGE-A9.

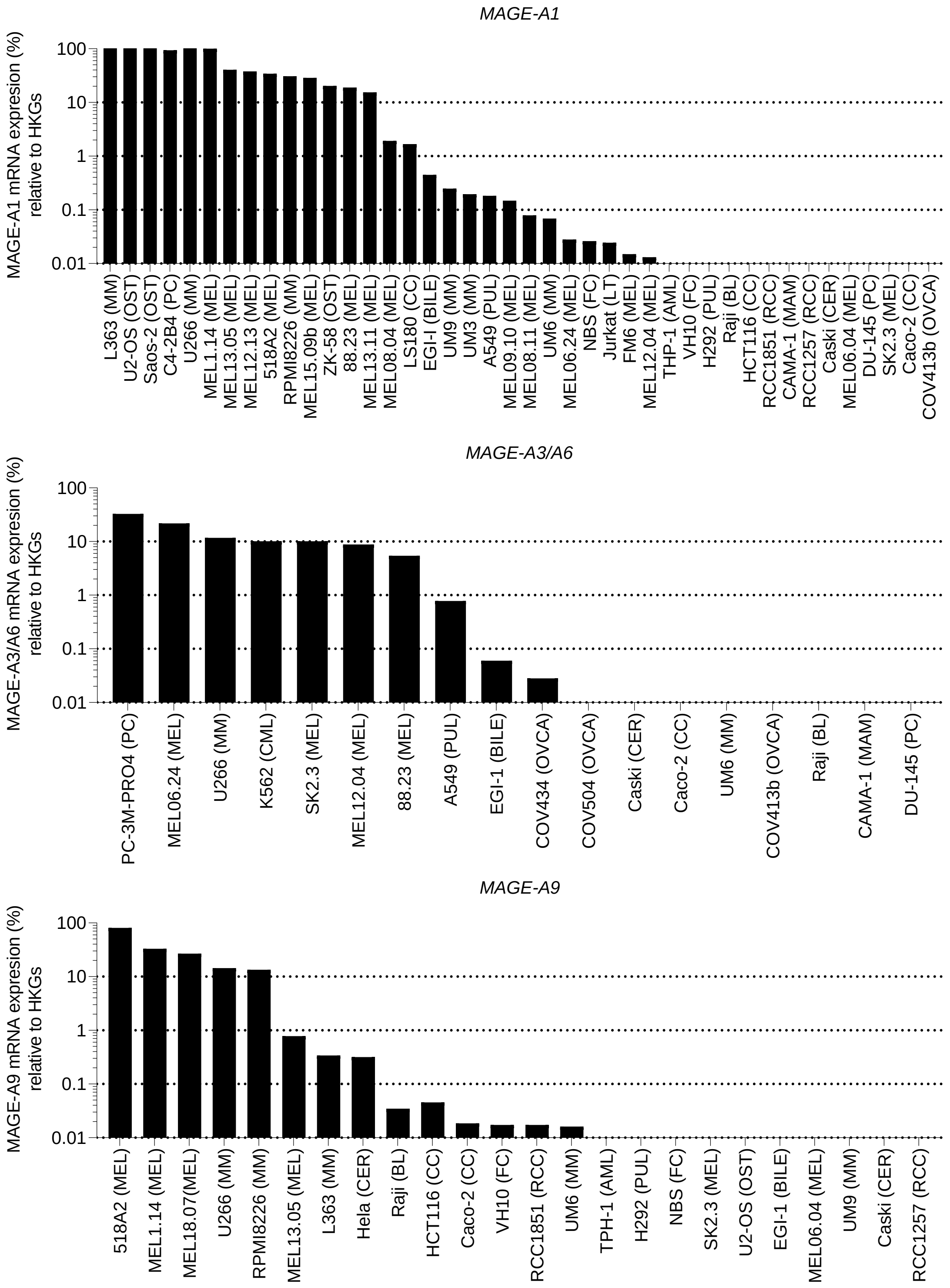

Figure S2. Target gene expression levels of *MAGE-A1*, *MAGE-A3/A6* and *MAGE-A9* mRNA levels. The expression levels were measured by qPCR for multiple cell lines with different origin including; multiple myeloma (MM), melanoma (MEL), prostate carcinoma (PC), fibroblast (FC), Burkitt lymphoma (BL), renal cell carcinoma (RCC), ovarium carcinoma (OVCA), colon carcinoma (CC), cervical carcinoma (CER, pulmonary carcinoma (PUL), leukemic T-cell lymphoblast (LT), bile duct carcinoma (BILE), osteosarcoma (OST), acute myeloid leukaemia (AML), chronic myeloid leukaemia (CML) cell lines and mammary carcinoma (MAM). Gene expression >0.01 is depicted in the graph relative to housekeeping genes (HKG). Values <0.01 relative gene expression are not depicted. *MAGE-A3* and *MAGE-A6* gene expression could not be discriminated from each other by qPCR.

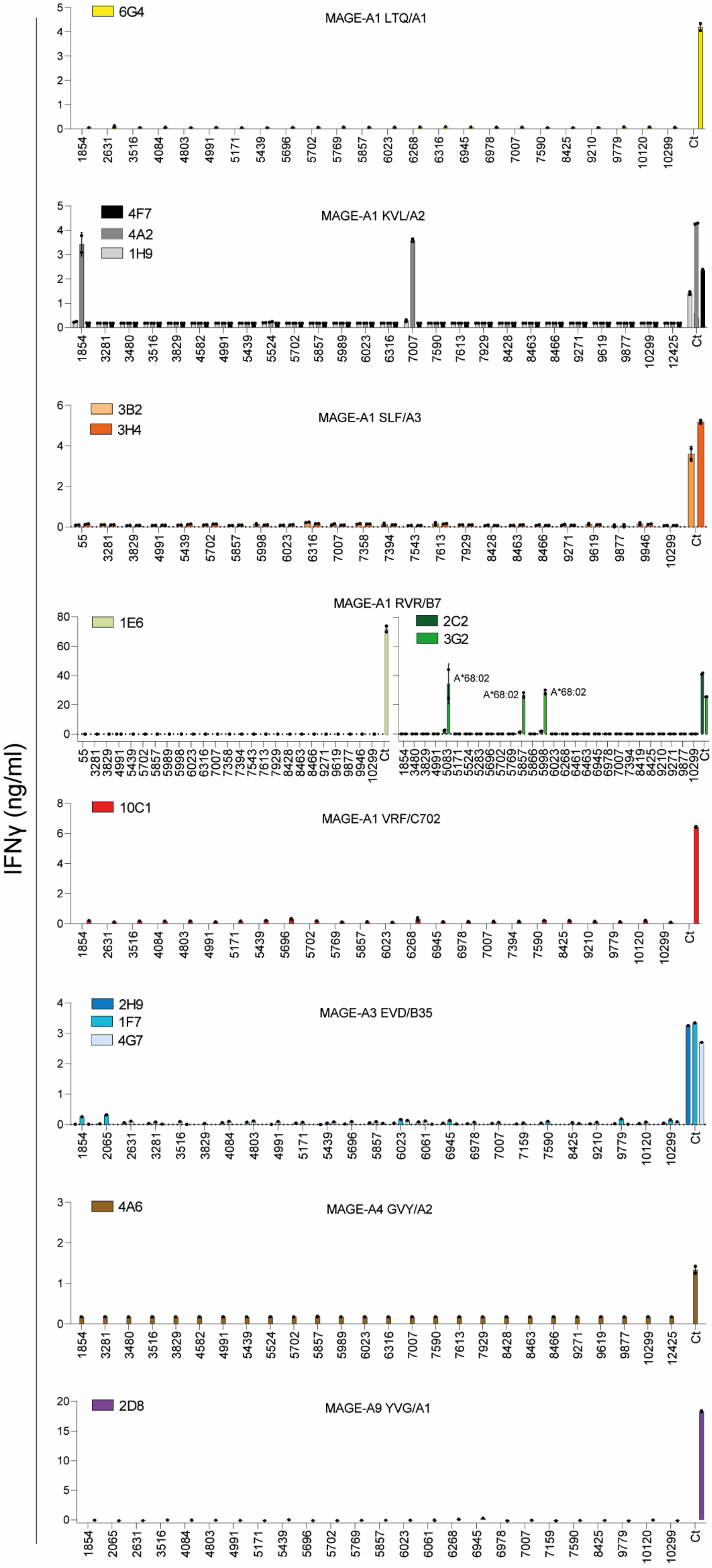

IFNγ (ng/ml)
MAGE-A1 LTQ/A1
6G4
MAGE-A1 KVL/A2
4F7
4A2
1H9
MAGE-A1 SLF/A3
3B2
3H4
MAGE-A1 RVR/B7
1E6
2C2
3G2
A*68:02
MAGE-A1 VRF/C702
10C1
MAGE-A3 EVD/B35
2H9
1F7
4G7
MAGE-A4 GVY/A2
4A6
MAGE-A9 YVG/A1
2D8
Ct

Figure S3. EBV-LCL panels to determine off-target reactivity against other peptide-HLA complexes. T cell clones were overnight stimulated with multiple EBV-LCLs. These EBV-LCL panels comprised of different EBV-LCLs expressing all HLA alleles with a prevalence of >1% in the Caucasian population. Peptide loaded HLA Td Raji cells were included as positive control (Ct) for T-cell function. The 4A2 clone (MAGE-A1 KVL/A2) demonstrated reactivity against 2 EBV-LCLs, the HLA restriction of this reactivity could not be identified, and therefore this T cell clone was excluded from further analyses. The 3G2 clone (MAGE-A1 RVR/B7) demonstrated reactivity against the HLA-A*68:02 positive EBV-LCLs. Values and error bars represent mean and standard deviations of technical duplicates.

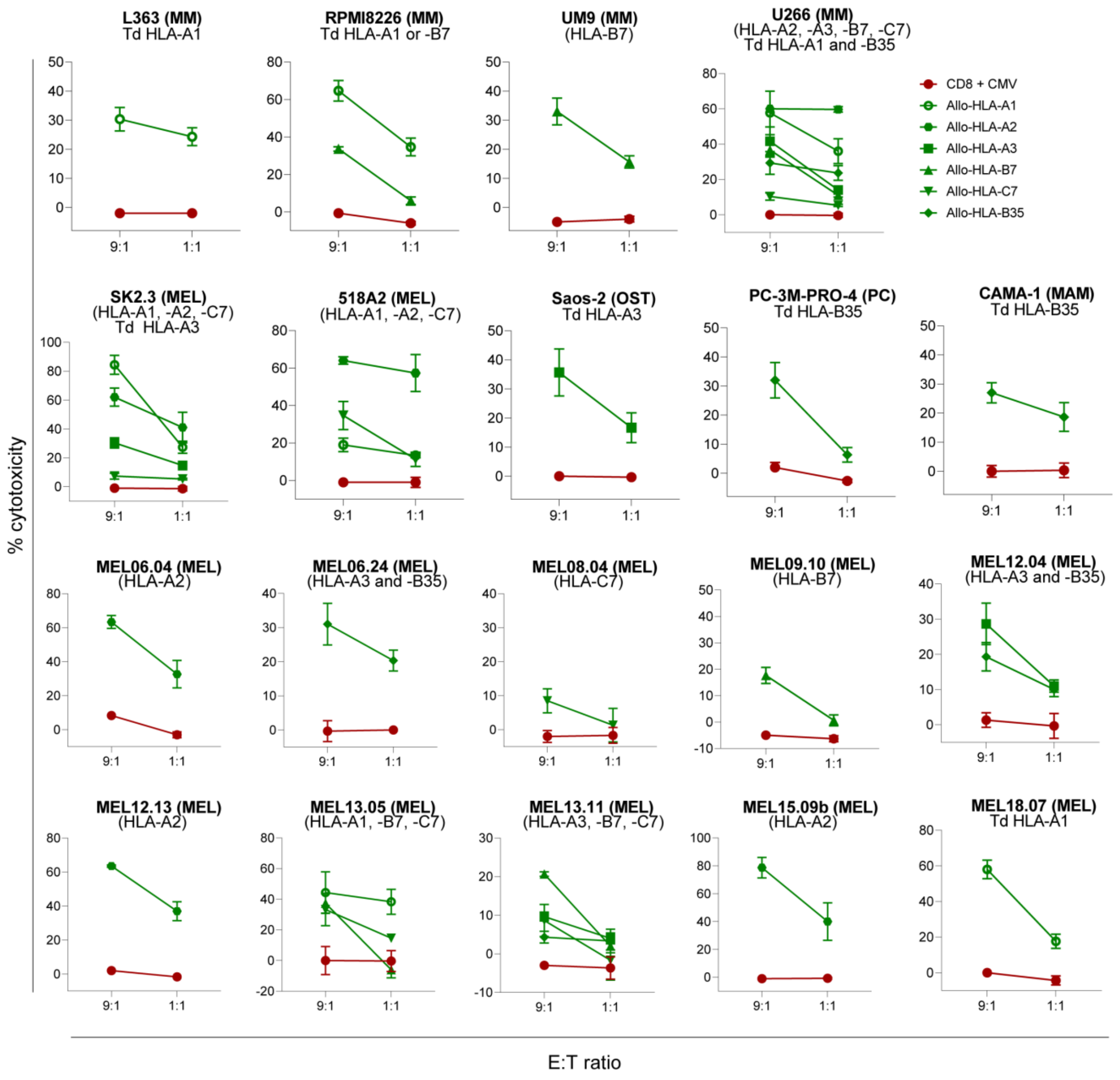

Figure S4. Positive and negative controls included for each target in the cytotoxicity assays. CMV TCR-T cell was included as a negative control (red) and an allo-HLA reactive T cell clone against the HLA of interest as a positive control (green). The tumor cell lines included were; Multiple myeloma (MM), melanoma (MEL), osteosarcoma (OST), prostate carcinoma (PC), and mammary carcinoma (MAM). Transduced HLA alleles are depicted in the graphs and naturally expressed HLA alleles are depicted between brackets. Cytotoxicity was assessed by an 6-hour ^{51}Cr-release assay with E:T ratio 9:1 and 1:1. Technical triplicates are depicted in the graph and the values are a representatives of at least two independent experiments.

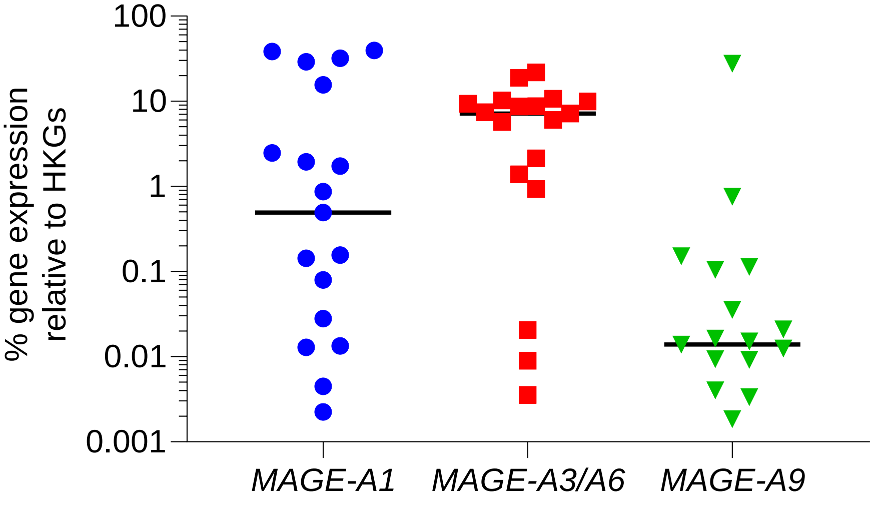

Figure S5. *MAGE-A1, MAGE-A3/A6* and *MAGE-A9* mRNA expression levels of early passage melanomas (passage ≤10) as measured by qPCR. *MAGE-A3* and *MAGE-A6* gene expression could not be discriminated from each other by qPCR. Gene expression >0.001% is depicted in the graph.

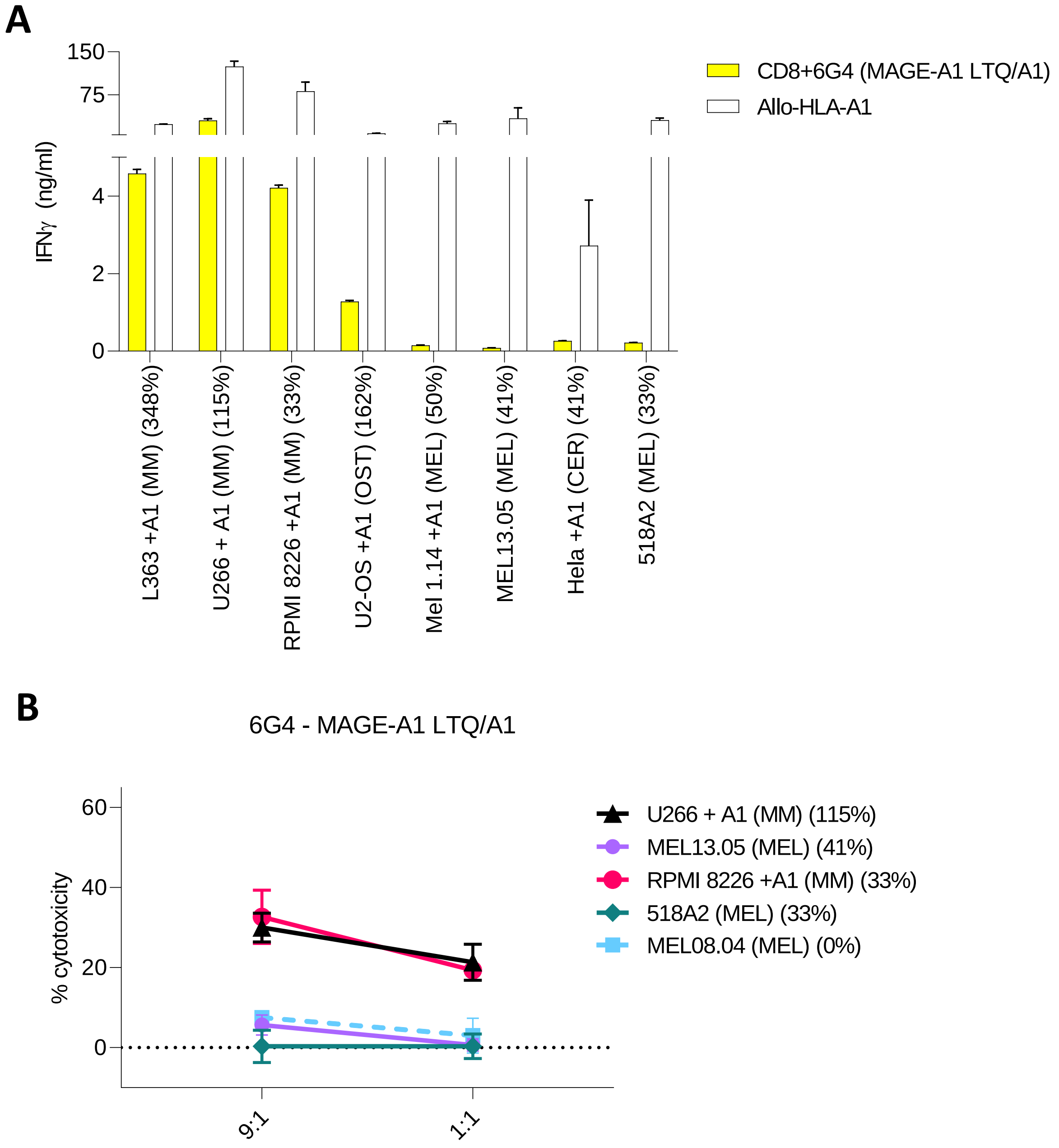

Figure S6. Reactivity of the 6G4 (MAGE-A1 LTQ/A1) TCR-T cells against tumor cell lines. (A) The 6G4 (MAGE-A1 LTQ/A1) TCR-T cell were overnight stimulated with MAGE-A1 expressing tumor cell lines of different origin including; melanoma (MEL), multiple myeloma (MM), cervical carcinoma (CER) and osteosarcoma (OST) cell lines. An allo-HLA reactive T cell clone was included to confirm proper HLA expression and recognition capacity of the targets. *MAGE-A1* gene expression levels, measured by qPCR, are depicted between brackets as percentage relative to housekeeping genes. Recognition was determined by IFN-γ production as measured by ELISA. Values and error bars represent mean and standard deviations of technical duplicates. (B) Cytotoxicity was assessed by a 6-hour ^{51}Cr-release assay at E:T ratio 9:1 and 1:1. Technical triplicates are depicted in the graph and the values are a representatives of at least two independent experiments.

**Table S1. Peptide-HLA complexes and selected MAGE-specific T cell clones**							
					**Recognition pattern identified T cell clones**		
**Target peptide**	**HLA-restriction**	**Target gene**	**Eluted from**	**NetMHC[A]**	**Td gene[B]**	**Potent & safe[C]**	**Final[D]**
ALIEVGPDHFC	A*02:01	*MAGE-C2*	OvaL10	7206	-	-	
ALKDVEERV	A*02:01	*MAGE-C2*	-	470	1	-	
ALKLKVAEL	B*08:01	*MAGE-A9*	RPMI8226 +B8	71	-	-	
ALREEEEGV	A*02:01	*MAGE-A1*	U266, C4-2B4, OvaL11	825	1	-	
EADPTGHSY	A*01:01	*MAGE-A1*	-	42	2	-	
EVDPIGHLY	A*01:01/<br>B*35:01	*MAGE-A3*	-<br>-	11<br>297	7<br>6	-<br>3	-<br>1(2H9)
FPSLREAAL	B*07:02	*MAGE-A1*	U266 and OvaL11	9	2	-	
FVYGEPREL	A*02:01	*MAGE-C2*	U266	512	3	-	
GLLGDNQIMPK	A*03:01	*MAGE-A1/A3/A6*	U266, RPMI8226 +A3	206	3	-	
GVYAGREHFV	A*02:01	*MAGE-C2*	U266	975	2	-	
GVYDGREHTV	A*02:01	*MAGE-A4*	U266, UM9 +A2, OvaL10	1752	1	-	
IMPKAGLLII	A*24:02	*MAGE-A3*	OvaL1	413	-	-	
IVLGVILTK	A*03:01	*MAGE-A9*	RPMI8226 +A3	27	-	-	
KIWEELSVLEV	A*02:01	*MAGE-A3/A6*	U266	87	-	-	
KVLEFLAKL	A*02:01	*MAGE-C2*	U266	25	3	-	
KVLEYVIKV	A*02:01	*MAGE-A1*	U266	7	18	3	1 (4F7)
LTQDLVQEKYLEY	A*01:01	*MAGE-A1*	RPMI8226 +A1	126	1	1	1 (6G4)
LVFGIELMEV	A*02:01	*MAGE-A3*	-	41	6	-	
RCFPVIFGK	A*03:01	*MAGE-A4*	U266, UM9 +A3	217	-	-	
RPADLTRVIM	B*07:02	*MAGE-A11*	Oval10	9	-	-	
RVRFFFPSL	B*07:02	*MAGE-A1*	U266	75	61	3	1 (3G2)
RVRFFFPSLR	A*03:01	*MAGE-A1*	Prediction	46	-	-	
RVRIAYPSL	B*07:02	*MAGE-A4*	U266	53	-	-	
RVRIAYPSLR	A*03:01	*MAGE-A4*	U266	120	7	-	
SLFRAVITK	A*03:01	*MAGE-A1*	U266	14	39	2	1 (3H4)
SMLGDGHSMPK	A*03:01	*MAGE-A9*	RPMI8226 +A3	68	-	-	
SVMGVYVGK	A*03:01	*MAGE-A9*	RPMI8226+A3	29	-	-	
TLDEKVAEL	A*02:01	*MAGE-C2*	U266	19	2	-	
TQDLVQEKY	A*01:01	*MAGE-A1/B1/B4*	RPMI8226 +A1	562	-	-	
VAELVHFLL	A*24:02	*MAGE-A3*	-	7689	1	-	
VIWEVLNAV	A*02:01	*MAGE-C2*	-	11	5	-	
VLGEEQEGV	A*02:01	*MAGE-A9*	RPMI8226 +A2	167	-	-	
VRFFFPSL	C*07:01/<br>C*07:02	*MAGE-A1*	Prediction/<br>U266	8807<br>5858	2<br>13	-<br>1	-<br>1(10C1)
YPSLREAAL	B*07:02	*MAGE-A4*	U266, UM9	7	-	-	
YVGKEHMF	A*24:02	*MAGE-A9*	RPMI8226 +A24	3852	-	-	
YVGKEHMFY	A*01:01	*MAGE-A9*	RPMI8226 +A1	341	1	1	1 (2D8)

MAGE specific peptides were identified form multiple myeloma cell lines U266, UM9 and RPMI8226; prostate cancer cell line C4-2B4, and primary ovarium carcinoma samples OvaL1, OVal10, and Oval11.

[A]Predicted NetMHC 4.0 affinity (nM)

[B]T cell clones produce IFN-γ after stimulation with MAGE-gene transduced Raji cell.

[C]T cell clones that show potent and strict recognition against Tumor cell line panels, EBV-LCL panel and MAGE-A panel.

[D]The final selection of most promising T cell clones for TCR gene therapy

Table S2. HLA-typing of EBV-LCLs used in EBV-LCL screenings			
**EBV-LCL**	**HLA-A**	**HLA-B**	**HLA-C**
55	01 – 68	08 – 53	04 - 07
1854	02:01 - 30:02	15:01 - 39:01	03:03 - 12:03
2065	01:01 - 02:01	37:01 - 39:01	06:02 - 07:02
2631	02:01 - 03:01	44:02 - 57:01	06:02 - 07:04
3281	01:01 - 32:01	08:01 - 45:01	06:02 - 07:01
3480	26:01 - 01:01	38:01 - 18:01	12:03 - 07:01
3516	03:01 - 26:01	07:02 - 14:01	07:02 - 08:02
3829	01:01 - 68:01	44:02	05:01 - 07:04
4084	03:01- 30:01	07:02 - 38:01	07:02 - 12:03
4582	01 - 30:01	08:01 -40:01	03:04 - 07:01
4803	03:01 - 33:01	07:02 - 14:02	07:02 - 08:02
4991	26:01 - 31:01	14:01 - 49:01	07:01 - 08:02
5171	02:01 - 66:01	40:01 - 41:02	03:04 - 17
5283	24:02 - 66:01	14:02 - 39:06	07:02 - 08:02
5524	02:01 - 31:01	15:01 - 15:17	03:04 - 07:01
5439	03:01 - 25:01	15:17 - 18:01	07:01 - 12:03
5696	02:05	58:01	unknown
5702	32:01 - 68:01	35:03 - 52:01	12:02 - 12:03
5769	02:01 - 68:01	35:03 - 37:01	04:01 - 06:02
5857	30:04 - 68:02	38:01 - 55:01	03:03 - 12:03
5866	02:01 - 11:01	35:01 - 51:01	04:01 - 14:02
5989	11:01 -68:01	38:01 - 39:01	07:02 - 12:03
6023	03:01 - 11:01	40:02 - 56:01	01:02 - 02:02
6268	02:01 - 24:02	35:02 - 44:02	04:01 - 05:01
6316	29:02 - 30:01	13:02 - 44:03	06:02 - 16:01
6461	02:01	40:02	02:02
6463	02:01	57:01	06:02
6945	03:01 - 25:01	18:01 - 35:01	04:01 - 12:03
6978	02:01 - 02:05	15:01 - 45:01	01:02 - 06:02
7007	02:05 - 29:02	27:05 - 44:03	01:02 - 16:01
7159	02:01	13:02 - 44:02	05:01 - 06:02
7394	01:01 - 32:01	35:08	04:01
7590	24:02 - 31:01	07:02 - 35:08	04:01 - 07:02
7613	01:01 - 24:02	15:01 - 37:01	03:03 - 06:02
7929	01:01	35:02- 52:01	04:01 - 12:02
8419	02:01 - 01:01	50:01 - 07:02	07:02 - 06:02
8425	23:01 - 02:01	41:01 - 40:01	17:01 - 03:04
8428	11:01 - 01:01	57:01 - 35:01	06:02 - 04:01
8463	11:01 - 01:01	51:01 - 50:01	15:02 - 06:02
8466	23:01	51:01 - 41:01	17:01 - 15:02
9210	02:01	15:01 - 51:01	03:03 - 15:02
9271	33:01 - 66:01	58:01 - 58:02	03:02- 06:02
9619	01:01 - 33:03	44:03 - 51:01	07:06 - 14:02
9877	01:01 - 23:01	08:01 - 41:02	07:01 - 17:03
9779	02:01 - 03:01	08:01 - 50:01	06:02 - 07:01
10120	02:01 - 24:02	07:02 - 40:02	02:02 - 07:02
10299	02:01 - 11:01	44:05 - 51:01	02:02 - 14:02
12425	23:01 - 36:01	15:03 - 53:01	02:10 - 04:01

Table S3. Primers included in the RT-qPCR experiments		
**Gene**	**Forward primer**	**Reverse primer**
MAGE-A1	GAGTCCTTGTTCCGAGCAGT	GGCTCCCTGGCTCGATATTT
MAGE-A3/A6	CCTGAGCAACGAGCGACG	TCAGAACCTTGCCTCCTCACC
MAGE-A9	GATCCTGCGCACTACGAGTT	ATGGGTAGCAGATGGGCTCT
GUSB	ACTGAACAGTCACCGACGAG	GGAACGCTGCACTTTTTGGT
PSMB4	GTTTCCGCAACATCTCTCGC	CATCAATCACCATCTGGCCG
VPS29	TGAGAGGAGACTTCGATGAGAATC	TCTGCAACAGGGCTAAGCTG
